# Computation of stagnation coating flow of electro-conductive ternary Williamson hybrid $\mathrm{GO}-\mathrm{AU}-{\mathrm{Co}}_{3}{\mathrm{O}}_{4}/\mathrm{EO}$ nanofluid with a Cattaneo–Christov heat flux model and magnetic induction

**DOI:** 10.1038/s41598-023-37197-8

**Published:** 2023-07-06

**Authors:** K. Bhagya Swetha Latha, M. Gnaneswara Reddy, D. Tripathi, O. Anwar Bég, S. Kuharat, Hijaz Ahmad, Dilber Uzun Ozsahin, Sameh Askar

**Affiliations:** 1 Department of Mathematics, Acharya Nagarjuna University Campus, Ongole, AP 523 001 India; 2 Department of Mathematics, National Institute of Technology, Uttarakhand, 246174 India; 3 Multi-Physical Engineering Sciences Group, Department of Mechanical and Aeronautical Engineering, Corrosion/Coatings Lab, Salford University, 3-08, SEE Building, Manchester, M54WT UK; 4 Section of Mathematics, International Telematic University Uninettuno, Corso Vittorio Emanuele II, 39, 00186 Rome, Italy; 5 Near East University, Operational Research Center in Healthcare, TRNC Mersin 10, 99138 Nicosia, Turkey; 6 Department of Computer Science and Mathematics, Lebanese American University, Beirut, Lebanon; 7 Department of Medical Diagnostic Imaging, College of Health Sciences, Sharjah University, Sharjah, United Arab Emirates; 8 Research Institute for Medical and Health Sciences, University of Sharjah, Sharjah, United Arab Emirates; 9 Department of Statistics and Operations Research, College of Science, King Saud University, P.O. Box 2455, Riyadh, 11451 Saudi Arabia

**Keywords:** Mathematics and computing, Nanoscience and technology, Physics

## Abstract

Modern smart coating systems are increasingly exploiting functional materials which combine multiple features including rheology, electromagnetic properties and nanotechnological capabilities and provide a range of advantages in diverse operations including medical, energy and transport designs (aerospace, marine, automotive). The simulation of the industrial synthesis of these multi-faceted coatings (including stagnation flow deposition processes) requires advanced mathematical models which can address multiple effects simultaneously. Inspired by these requests, this study investigates the interconnected magnetohydrodynamic non-Newtonian movement and thermal transfer in the Hiemenz plane's stagnation flow. Additionally, it explores the application of a transverse static magnetic field to a ternary hybrid nanofluid coating through theoretical and numerical analysis. The base fluid (polymeric) considered is engine-oil (EO) doped with graphene $\left(GO\right)$, gold $\left(Au\right)$ and Cobalt oxide $\left(C{o}_{3}{O}_{4}\right)$ nanoparticles. The model includes the integration of non-linear radiation, heat source, convective wall heating, and magnetic induction effects. For non-Newtonian characteristics, the Williamson model is utilized, while the Rosseland diffusion flux model is used for radiative transfer. Additionally, a non-Fourier Cattaneo–Christov heat flux model is utilized to include thermal relaxation effects. The governing partial differential conservation equations for mass, momentum, energy and magnetic induction are rendered into a system of coupled self-similar and non-linear ordinary differential equations (ODEs) with boundary restrictions using appropriate scaling transformations. The dimensionless boundary value problem that arises is solved using the bvp4c built-in function in MATLAB software, which employs the fourth-order Runge–Kutta (RK-4) method. An extensive examination is conducted to evaluate the impact of essential control parameters on the velocity $f{^{\prime}}\left(\zeta \right)$, induced magnetic field stream function gradient $g{^{\prime}}\left(\zeta \right)$ and temperature $\theta \left(\zeta \right)$ is conducted. The relative performance of ternary, hybrid binary and unitary nanofluids for all transport characteristics is evaluated. The inclusion of verification of the MATLAB solutions with prior studies is incorporated. Fluid velocity is observed to be minimized for the ternary $\mathrm{GO}$–$\mathrm{Au}$–${\mathrm{Co}}_{3}{\mathrm{O}}_{4}$ nanofluid whereas the velocity is maximized for the unitary cobalt oxide $\left({\mathrm{Co}}_{3}{\mathrm{O}}_{4}\right)$ nanofluid with increasing magnetic parameter ($\beta ).$ Temperatures are elevated with increment in thermal radiation parameter (*Rd*). Streamlines are strongly modified in local regions with greater viscoelasticity i.e. higher Weissenberg number $(We)$. Dimensionless skin friction is significantly greater for the ternary hybrid $GO$–$Au$–$C{o}_{3}{O}_{4}/EO$ nanofluid compared with binary hybrid or unitary nanofluid cases.

## Introduction

Stagnation-point flows have prominent applications in engineering and industrial fields such as hybrid reactors, metallurgical processing, materials fabrication, particle deposition and external plasma aerodynamics. They also arise in geophysics in atmospheric transport, for example when airflow stagnates on the upstream face of a mountain ridge. Stagnation flows can be delineated into plane stagnation (Hiemenz) and radial (Homann) types. In the former, which are more common in materials fabrication operations^1^, the impinging jet of fluid on a flat substrate (surface being coated) assumes an axisymmetric flow structure at a right angle to it and then flows away radially in all directions. Laminar plane stagnation flows allow the Navier–Stokes equations to be reduced to much simpler forms enabling a wide range of solutions to be generated. This regime is encountered in many important applications in modern manufacturing technology including combustion reactors^2^, catalysis^3^, polymer adsorption processes^4^ and blade coating dynamics^5^. In recent years, materials science developments have expanded considerably. New smart functional materials have been produced which combine multiple properties to yield intelligent designs that can accommodate a variety of loading scenarios and achieve enhanced durability in the field. These materials respond to electrical and magnetic fields, light, sound, chemical reactions and so on. They are designed also to combat corrosion, bacterial infection and surficial damage from extreme heat and often feature optical-reactive elements. Many novel materials have emerged largely due to the intrusion of nanotechnology into the manufacturing sector. These include thermal-sprayed functional magnetic coatings^6^, micro/nano-structured electromagnetic sensor films modified with laser processing^7^, multi-layered nanostructured thin coating layers^8^, electroconductive polymers (ECPs)^9^ and multi-compositional nanocoatings^10,11^. Many of these materials have made profound improvements in the biomedical and marine engineering sectors. To simulate the fabrication of these adaptive materials, a combination of a number of areas of fluid mechanics, electromagnetics, rheology and thermal sciences is required. Stagnation flows by their nature feature boundary layer behaviour. Historically early work in magnetohydrodynamic (MHD) stagnation flows was motivated by aerospace developments, in particular flow at the nose of a bluff body (rocket). Rossow^12^ presented the first comprehensive study of external boundary layer stagnation point flow and heat transfer in Newtonian electrically conducting gases. Using a similarity approach, he derived analytical solutions for the influence of the Hartmann number (magnetic body force parameter) on transport characteristics. Subsequently Gribben^13^ generalized the Rossow model using asymptotic expansions. Katagari^14^ considered perforations at the boundary in magnetohydrodynamic boundary layer flow with wall transpiration. He used an integral formulation and numerical quadrature to obtain robust solutions for velocity, skin friction, displacement thickness and momentum thickness, although he neglected heat transfer effects. Mahapatra and Nandy^15^ examined the impact of radiative-convective stagnation flow on a contracting sheet with suction and injection effects. They demonstrated the existence of multiple solutions for the boundary layer equations when the shrinking velocity to free stream velocity ratio reaches a critical value, and these solutions are notably influenced by the suction/injection effect. These investigations were confined to Newtonian fluids. However, many advanced functional coatings including electro-conductive polymers and magnetorheological (MR) liquids^16,17^ exhibit non-Newtonian characteristics. These require appropriate rheological formulations to more accurately represent the shear stress–strain behaviour, which is not achievable with the classical Newtonian model (Navier–Stokes equations). Largely motivated by polymeric functional materials processing applications, a number of researchers have therefore explored a variety of non-Newtonian models in stagnation point coating fluid mechanics simulations. In their study, Mahapatra et al.^18^ investigated the behaviour of hydromagnetic convective stagnation-point flow. The study focused on the movement of a non-Newtonian viscoelastic fluid over a flat deformable surface. The surface was subjected to stretching within its own plane, with the stretching rate being proportional to the distance from the stagnation point. The researchers employed the Walters' Bʹ shoart memory model and observed the formation of an inverted boundary layer when the surface stretching velocity surpassed the velocity of the free stream. They also showed that strong heating is induced with greater magnetic field whereas flow deceleration is produced. Hayat et al*.*^19^ examined the magneto-convective stagnation flow of a Cross fluid (with viscosity dependence on shear rate) from an extending wall with a Runge–Kutta–Fehlberg method. The researchers noted that an increase in the Weissenberg number (indicating a stronger viscoelastic effect) resulted in the generation of flow retardation, while the Hartmann magnetic number led to an increase in the thickness of the thermal boundary layer. Gupta et al*.*^20^ used Eringen’s micropolar rheological model and a variational finite element method to compute the magnetohydrodynamic stagnation point convective boundary layer flow from an isothermal stretching sheet. They showed that local Nusselt number is reduced with magnetic field whereas skin friction is elevated with micropolar vortex viscosity parameter (Eringen number). Further studies include Gorla^21^ who deployed a power-law model and Sarkar and Sahoo^22^ who utilized the second grade Reiner–Rivlin viscoelastic model. A finite element study of rotational convective boundary layer of a UCM non-Newtonian fluid with heat transfer was presented by Khan et al.^23^.

The above magnetohydrodynamic flow studies generally neglected induced magnetic field effects. The applied magnetic field in materials processing however can generate magnetic induction which creates a separate magnetic boundary layer distinct from the velocity and thermal boundary layers. The induced magnetic field is usually aligned with the streamwise direction i.e. it is perpendicular to the applied magnetic field and can dramatically modify transport characteristics in coating regimes. Magnetic induction in boundary layer flows was first addressed theoretically by Glauert^24^ obtained power series solutions encompassing a wide range of electrical conductivity parameter values, including both large and small values, although he did not consider heat transfer. He identified that when the applied magnetic field intensity, exceeds a critical value, boundary-layer separation is induced. Takhar et al*.*^25^ generalized Glauert’s analysis to consider unsteady heat transfer from an impulsively moving wall. They used a finite difference technique and showed that with increasing inverse magnetic Prandtl number (ratio of the magnetic to viscous diffusivity) there is a strong elevation in induced magnetic field at the surface, Nusselt number, and also surface skin friction owing to a reduction in electrical conductivity and weaker influence of boundary layer velocity over the magnetic lines of forces. More recently Ali et al*.*^26^ have studied magnetic induction effects with nonlinear convection from a stretching wall. Akter et al*.*^27^ have considered thermal radiation flux effects with a spectral relaxation numerical scheme. Numerous investigations have validated the noteworthy alterations observed in velocity and temperature properties caused by the influence of induced magnetic fields. The Fourier model is the conventional approach employed in heat transfer analysis for thermal conduction. This model is parabolic in nature and incorrectly assumes infinite thermal waves since it neglects thermal relaxation effects which can rise in certain materials processing operations. To provide a more robust formulation, the non-Fourier heat flux model, which is hyperbolic, has been introduced and accommodates finite waves in heat conduction^28^. Also known as the Cattaneo–Christov heat flux model, this formulation has been implemented in many flow scenarios including swirl coating^29^ and also in a number of studies of stagnation flows. Hayat et al*.*^30^ employed a homotopy method to compute the characteristics of a chemically reactive stagnation flow with non-Fourier heat and mass transfer in a Maxwell viscoelastic fluid surrounding a stretching cylinder. The researchers noted that when the Deborah number (the ratio of relaxation time to observation time) and the non-Fourier thermal relaxation parameter were increased, it resulted in a slowdown of the axial flow. Furthermore, they discovered that both the temperature and the thickness of the thermal boundary layer decreased due to the prolongation of heat transfer caused by finite thermal conduction. Mehmood et al*.*^31^ employed numerical techniques to calculate the non-Fourier reactive magnetohydrodynamic Oldroyd-B oblique stagnation flow from a stretching wall subjected to convective heating. They showed that increasing thermal relaxation (non-Fourier heat flux parameter) cools the regime and suppresses thermal boundary layer thickness and that flow acceleration is produced in the tangential direction whereas flow deceleration is induced in the normal direction with greater Deborah (viscoelastic) numbers. Further investigations of non-Newtonian non-Fourier stagnation flows have deployed the Maxwell model with quadratic convection^32^, the Eyring-Powell model for roll coating^33^, the Reiner-Rivlin third grade viscoelastic model^34^ and the tangent-hyperbolic shear thinning model^35^. All of these studies have verified the notable disparity in flow characteristics resulting from non-Fourier effects.

A substantial development in modern materials design has been achieved with the use of nanotechnology. Many complex materials can now be synthesized at the nanoscale level to provide a wider range of functionalities in for example coatings. An important category of these nanomaterials is nanofluids. The enhancement of thermal properties in a fluid (e. g. external coating films) is attainable by embedding nanoparticles in a base fluid to create nanofluid coatings. Choi^36^ pioneered nanofluid technology in the 1990s and proved that the thermal conductivity is remarkably enhanced by strategically suspending nanoparticles in regular base fluids. Metallic nanoparticles and their oxides have been extensively explored subsequently in smart functional nano-coating designs including nickel, cerium, gold, silver, titanium, copper, cobalt, zirconium, zinc and aluminium. Carbon-based nanoparticles and carbon nanotubes (CNTs) have also been investigated including silicon, graphene, graphite, silicon carbide etc.^37–40^. Unique performance can be achieved by careful selection of the nanoparticles for specific applications in engineering. In parallel with experimental investigations, many theoretical studies of nanofluid transport in manufacturing have been communicated, including stagnation flows. Numerous methodologies have been devised to replicate nanoscale properties, with some commonly employed techniques being the Tiwari-Das volume fraction model, Buongiorno's two-component model, and mixture models^41^. Mustafa et al*.*^42^ used a homotopy method and Buongiorno’s nanoscale model to derive analytical solutions, noting that both the local Nusselt and Sherwood numbers increase with Brownian motion, thermophoresis, Prandtl and Lewis numbers. The stagnation point nanofluid flow behaviour from a stretching sheet with the Tiwari-Das nanoscale model was explored by Bachok et al.^43^, who considered three types of nanoparticles. They noted that the fluid temperature is greatest with alumina (${Al}_{2}{O}_{3}$) nanoparticles. Bachok et al*.*^44^ examined the characteristics of stagnation point flow using a copper–water nanofluid. Other studies include Nadeem et al*.*^45^ on non-orthogonal stagnation point flow of CNT-engine oil nanofluids on a convectively heated stretching sheet, Additional investigations of magneto-convective Hiemenz nanofluid flows include Farooq et al*.*^46^ (who considered radiative heat transfer and viscoelastic behaviour) and Shukla et al*.*^47^ (who examined entropy generation, time-dependent and wall transpiration effects). Magnetic induction effects were investigated for unitary nanofluids by Ferdows et al*.*^48^ who computed also shape factor effects for elliptic single-/multi-wall carbon nanotubes and Iqbal et al*.*^49^ who also considered gyrotactic micro-organisms suspended in the electrically conducting nanofluid. These studies demonstrated that generally induced magnetic field is enhanced with nanoparticle volume fraction.

Until quite recently, the vast majority of nanofluid dynamic simulations reported in the literature were restricted to unitary nanofluid (mono nanofluid) in which only a single nanoparticle type (metallic or carbon-based) was studied. However, engineers subsequently explored the combination of multiple nanoparticles in the same nanofluid and identified that further enhancement in thermal conductivity, viscosity modification and heat transfer performance is possible with these *hybrid* nanofluids. O’Scott et al*.*^50^ reviewed the developments in hybrid nanofluids recently with a focus on materials processing operations. They confirmed that hybrid nanofluids generally produce superior thermal and rheological behaviour compared with mono-nanofluids, largely due to the synergistic effect of different nanomaterials which assist each other in improving global characteristics of the hybrid nanofluids. As such multiple nanoparticles can amalgamate the chemical and physical properties of different nanoparticles simultaneously and still sustain a stable, homogeneous condition during operations. Recent studies of dual nanoparticle hybrid nanofluids have assessed a variety of combinations of metallic nanoparticles and their oxides. These include Bhatti et al*.*^51^ who computed the performance of magnesium-nickel oxide nanoparticles in aqueous base fluids for novel solar collector magnetic coatings. Prakash et al*.*^52^ considered titania, alumina or copper metallic nanoparticles in electro-osmotic bio-micro-fluidic pumping systems. Prakash et al*.*^53^ further appraised the relative performance of hybrid (Ag–Al_2_O_3_) nanofluids and Al_2_O_3_ unitary nanofluid with ethylene glycol (EG) base fluid. Ghandi et al*.*^54^ compared the efficacy of nanoparticle drug delivery using unitary gold nanoparticles and hybrid magnetic $\mathrm{Au}-{\mathrm{Al}}_{2}{\mathrm{O}}_{3}/\mathrm{Blood}$ Au–Al_2_O_3_ nanoparticles. Bhatti et al*.*^55^ studied gold versus gold-magnesium oxide hybrid nanoparticles in electromagnetic thermal duct pumping flows.

In all these investigations the hybrid (dual) nanofluid achieved significantly better results than the unitary nanofluids. As a result of the success of dual hybrid nanofluids, engineers have further expanded this technology to consider triple nanoparticle designs. These are known as *ternary hybrid nanofluids* and offer yet greater potential in many sectors including biomedicine, energy and power and functional coating manufacture. In such applications, three dissimilar nanoparticles are disseminated in the base fluid and have been shown to produce the best improvements in dynamic viscosity and thermal conductivity and substantially greater stability than dual hybrid nanofluids or unitary nanofluids. Qayyaum et al*.*^56^ have studied carbon nanotube hybrid nanofluid transport between spinning and radially stretching disks with chemical reaction effects. Recent simulations deploying ternary hybrid nanofluids include Mahmood et al*.*^57^ who addressed Cu–Fe_3_O_4_–SiO_2_/polymer ternary nanofluid hydromagnetic stagnation flow from an extending/contracting porous-walled cylinder. Algehyne et al*.*^58^ scrutinized the performance of titanium dioxide (TiO_2_)-cobalt ferrite (CoFe_2_O_4_)-magnesium oxide (MgO) nanocomposite base hybrid nanofluids in magnetized stagnation point flow from a stretching sheet with a non-Fourier heat flux model. Haneef et al*.*^59^ studied bi-directional flow from a plate with (*Al*_2_*O*_3_–*TiO*_2_–*SiO*_2_) ternary-water viscoelastic nanofluid. The non-Newtonian hyperbolic tangent magnetic nanofluid flow with tri-hybrid nanoparticles was investigated by Nazir et al*.*^60^. Nazir et al.^61^ have analyzed ternary Sisko rheological nano-fluids an in the presence of heat source. Sohail et al*.*^62^ have reported on pseudo-plastic (shear-thinning) tri-hybrid nanofluid transport from a stretching surface. Animasaun et al*.*^63^. Investigated the aluminum oxide ${Al}_{2}{O}_{3}$-silver $Ag$-aluminium $Al$-water ternary hybrid nanofluid flow from a convectively heated surface with magnetic induction. Other recent numerical and analytical methods^64–73^ can be used for such types of complex problems.

A close inspection of the literature has identified that thus far the Hiemenz plane stagnation point flow of electroconductive ternary non-Newtonian Williamson hybrid nanofluid (with engine oil base fluid) from a convective heated surface under transverse magnetic field, has not been addressed in any study. This is the focus of the present investigation. A non-Fourier Cattaneo–Christov heat flux model, non-linear radiation heat transfer and magnetic induction effects are also considered. The novelties of the present work, motivated by impinging flow on substrates encountered in the manufacture of smart magnetic nano-coatings^74^, is the therefore the simultaneous examination of ternary $\mathrm{GO}-\mathrm{AU}-{\mathrm{Co}}_{3}{\mathrm{O}}_{4}$ (graphene oxide-gold-cobalt oxide) nanoparticles in magnetic non-Newtonian oil base fluid with non-Fourier, thermal radiation and induced magnetic field effects. The governing partial differential conservation equations for mass, momentum, energy and magnetic induction are rendered into a system of coupled self-similar and non-linear ordinary differential equations (ODEs) with associated boundary conditions via appropriate scaling transformations. The RK-4 method, available as the bvp4c built-in function in MATLAB software, is employed to solve the dimensionless boundary value problem. An extensive analysis is carried out to evaluate the influence of essential control parameters on the dimensionless velocity $f{^{\prime}}\left(\zeta \right)$, induced magnetic field stream function gradient $g{^{\prime}}\left(\zeta \right)$ and temperature $\theta \left(\zeta \right)$ is conducted. The relative performance of ternary, hybrid binary and unitary nanofluids for all transport characteristics is evaluated. Validation of the MATLAB solutions with previous studies is included. Additionally, streamline contour plots are presented for the ternary $GO-AU-C{o}_{3}{O}_{4}/EO$ hybrid nanofluid.

## Mathematical model

**Figure 1:**
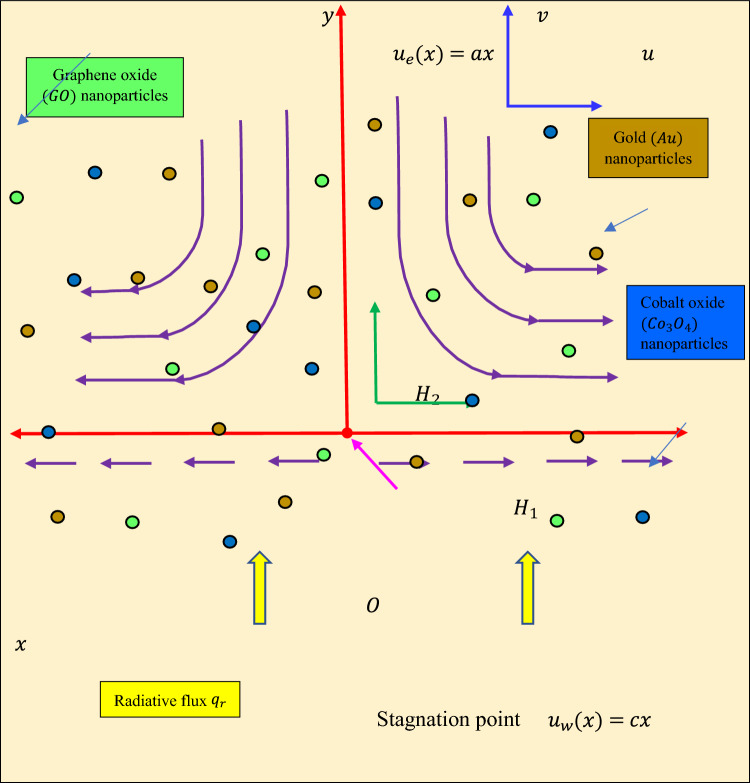
Physical model for smart nano-coating stagnation flow.

Steady, incompressible, two-dimensional stagnation flow of non-Newtonian Williamson Graphene oxide $\left(GO\right)$-Gold $\left(Au\right)$-Cobalt oxide $\left(C{o}_{3}{O}_{4}\right)$-engine oil $\left(EO\right)$ ternary hybrid nanofluid on a stretching sheet in an (*x, y*) coordinate system, with the stagnation point at the origin, *O*, is investigated. The physical model is shown schematically in Fig. 1. The $x$-axis is orientated along the stretching sheet and the electroconductive ternary hybrid $GO-AU-C{o}_{3}{O}_{4}/EO$ nanofluid occupies the region $y>0$.

There is no external electrical field and therefore the impact of polarization of charges is negligibly small. Ohmic dissipation, viscous dissipation, thermal stratification and thermal dispersion are also neglected. The velocities ${u}_{w}\left(x\right)=cx$ and ${u}_{e}\left(x\right)=ax$ are prescribed at the wall (stretching sheet) and in the free stream, where $a$ and $c$ are positive constants. The physical effects of non-linear radiation, Cattaneo–Christov heat flux and heat source are incorporated in the thermal energy equation. The non-Newtonian base fluid is assumed to be optically thick and absorbing, emitting but not reflecting thermal radiation. In addition, convective heating is considered at the wall. Magnetic Reynolds number is large enough for the magnetic flux lines to be advected with the fluid flow generating an induced magnetic field ${\varvec{H}}$ which has two components, ${H}_{1}$ and ${H}_{2}$ which denote the magnetic flux density along (parallel) and normal to the wall, respectively. Based on the aforementioned assumptions, the equations governing continuity, momentum, induced magnetic field, and heat can be formulated, by extending the models of Mustafa et al*.*^42^, Iqbal et al*.*^49^ and Animasaun et al*.*^63^, as follows^26,42,49,59,63^:

1 $$\frac{\partial u}{\partial x}+\frac{\partial v}{\partial y}=0$$

2 $$\frac{\partial {H}_{1}}{\partial x}+\frac{\partial {H}_{2}}{\partial y}=0$$

3 $$u\frac{\partial u}{\partial x}+v\frac{\partial v}{\partial y}+\frac{{\mu }_{e}{H}_{e}}{4\pi {\rho }_{thnf}}\frac{d{H}_{e}}{dx}=\frac{{\mu }_{e}{H}_{e}}{4\pi {\rho }_{thnf}}\frac{d{H}_{e}}{dx}\left({H}_{1}\frac{\partial {H}_{1}}{\partial x}+{H}_{2}\frac{\partial {H}_{2}}{\partial y}\right)+{u}_{e}\frac{\partial {u}_{e}}{\partial x}+\frac{{\mu }_{thnf}}{{\rho }_{thnf}}\frac{{\partial }^{2}u}{\partial {y}^{2}}+\sqrt{2}{v}_{f}\Gamma \frac{\partial u}{\partial y}\frac{{\partial }^{2}u}{\partial {y}^{2}}$$

4 $$u\frac{\partial {H}_{1}}{\partial x}+v\frac{\partial {H}_{2}}{\partial y}={H}_{1}\frac{\partial u}{\partial x}+{H}_{2}\frac{\partial u}{\partial y}+{\mu }_{e}\frac{{\partial }^{2}{H}_{1}}{\partial {y}^{2}}$$

5 $$u\frac{\partial T}{\partial x}+v\frac{\partial T}{\partial y}-{\varepsilon }_{t}\left[{u}^{2}\frac{{\partial }^{2}T}{\partial {x}^{2}}+2uv\frac{{\partial }^{2}T}{\partial x\partial y}+{v}^{2}\frac{{\partial }^{2}T}{\partial {y}^{2}}+u\frac{\partial u}{\partial x}\frac{\partial T}{\partial x}+v\frac{\partial u}{\partial y}\frac{\partial T}{\partial x}+u\frac{\partial v}{\partial x}\frac{\partial T}{\partial y}+v\frac{\partial v}{\partial y}\frac{\partial T}{\partial y}\right]={\alpha }_{thnf}\frac{{\partial }^{2}T}{\partial {y}^{2}}-\frac{1}{{\left(\rho {c}_{p}\right)}_{thnf}}\frac{\partial {q}_{r}}{\partial y}+\frac{{Q}_{0}}{{\left(\rho {c}_{p}\right)}_{thnf}}\left(T-{T}_{\infty }\right)$$

The associated boundary conditions^63^ are:

$$u={u}_{w}\left(x\right)=cx,v=0, \frac{\partial {H}_{1}}{\partial y}=0, {H}_{2}=0,-{k}_{thnf}\frac{\partial T}{\partial y}={h}_{f}\left({T}_{w}-T\right) \; at \; y=0$$

6 $$u\to {u}_{e}\left(x\right)=ax, { H}_{1}\to {H}_{e}\left(x\right)={H}_{0}x, T\to {T}_{\infty }\mathrm{\, as\, }y\to \infty$$

Here $u,v$ and ${H}_{1}, {H}_{2}$ denote the velocities and magnetic stream function components along $x$-axis and $y$-axis, ${v}_{f}$ is the kinematic viscosity of the engine oil base fluid, ${\mu }_{e}$ is the magnetic diffusivity, ${\rho }_{thnf}$ is the density of ternary nanofluid, ${\mu }_{thnf}$ is the dynamic viscosity of ternary nanofluid, $T$ is the nanofluid temperature, $\Gamma$ is the viscoelastic relaxation time, ${k}_{thnf}$ is the thermal conductivity of ternary hybrid nanofluid, ${q}_{r}$ is the radiative heat flux, ${Q}_{0}$ is the heat source, ${h}_{f}$ is the heat transfer coefficient, ${\alpha }_{thnf}$ is the thermal diffusivity, ${\varepsilon }_{t}$ is the non-Fourier thermal relaxation time and ${\left(\rho {c}_{p}\right)}_{thnf}$ is the specific heat capacitance of ternary hybrid $GO-AU-C{o}_{3}{O}_{4}/EO$ nanofluid. The thermo-physical properties of the three distinct nanoparticles considered in addition to the oil base fluid are presented in Table 1**.**

**Table 1:** The thermo-physical characteristics of Graphene oxide $\left(GO\right)$, Gold $\left(Au\right)$ and Cobalt oxide $\left(C{o}_{3}{O}_{4}\right)$ nanoparticles^46,60^.

Characteristics	$\rho$	$k$	${c}_{p}$	$\beta$	$\sigma$
$GO$	1800	5000	717	2.84 × 10^–4^	6.30 × 10^7^
$Au$	8908	91	445	0.0000134	1.7 × 10^7^
$C{o}_{3}{O}_{4}$	8862	99.2	421	1.85 × 10^–5^	1.85 × 10^–6^

The energy Eq. (5) features the radiative heat flux ${q}_{r}$. This can be modified via Rosseland’s approximation^46,75^ which is an absorbing, emitting but non-scattering uni-directional radiative diffusion model and is simulated by the following expression:

7 $${q}_{r}=-\frac{4}{3}\frac{{\sigma }^{*}}{{k}^{*}}\frac{\partial {T}^{4}}{\partial y}=-\frac{16}{3}\frac{{T}_{\infty }^{3}}{{k}^{*}}\frac{\partial T}{\partial y}$$

Here ${T}_{\infty }$ is free stream temperature, *σ*^∗^ is Stefan-Boltzmann radiation constant and k* is extinction coefficient. The primitive partial differential Eqs. (1)–(5) with boundary conditions (6) are challenging to solve, even numerically. It is judicious therefore to introduce by the following similarity transformation and variables, following Mansur et al.^76^:

$$u=cx, \frac{\partial f}{\partial \zeta }, v=\sqrt{c{\vartheta }_{f}}f\left(\zeta \right), \frac{\partial g}{\partial \zeta }= \frac{{H}_{1}}{{H}_{0}x} , {H}_{2}=-{H}_{0}\sqrt{\frac{{\vartheta }_{f}}{c}}g\left(\zeta \right),\zeta =y\sqrt{\frac{c}{{\vartheta }_{f}}},$$

$$A=\frac{a}{c}, Bi=\sqrt{\frac{{\vartheta }_{f}}{c}} \frac{{h}_{f}}{{k}_{f}}, \theta \left(\zeta \right)=\frac{T-{T}_{\infty }}{{T}_{w}-{T}_{\infty }}, \beta =\frac{{\mu }_{e}{H}_{0}^{2}}{4\pi {\rho }_{f}{c}^{2}}, {Pr}_{m}=\frac{{\vartheta }_{f}}{{\alpha }_{m}}, Pr=\frac{{\vartheta }_{f}}{{\alpha }_{f}},$$

$$We=x\Gamma \sqrt{\frac{{c}^{3}}{{\vartheta }_{f}}}, Q=\frac{{Q}_{0}}{c{\left(\rho {c}_{p}\right)}_{f}}, {T={T}_{\infty }\left(1+\left({\theta }_{w}-1\right)\theta \right), \theta }_{w}=\frac{{T}_{w}}{{T}_{\infty }},$$

8 $${\Gamma }_{t}={a\varepsilon }_{t}, Rd= \frac{16{\sigma }^{*}{T}_{\infty }^{3}}{3{k}^{*}{k}_{f}}$$

Invoking these transformations, the original partial differential boundary value problem is reduced to a self-similar, nonlinear coupled ordinary differential boundary value problem^59,63^:

9 $$\left(1+We{f}^{{^{\prime}}{^{\prime}}}\right){f}^{{^{\prime}}{^{\prime}}{^{\prime}}}+\frac{{N}_{2}}{{N}_{1}}\left[f{f}^{{^{\prime}}{^{\prime}}}-{{f}^{^{\prime}}}^{2}+\left(\beta {{g}^{^{\prime}}}^{2}-g{g}^{{^{\prime}}{^{\prime}}}-1\right)\right]+{A}^{2}=0,$$

10 $${g}^{{^{\prime}}{^{\prime}}{^{\prime}}}+{Pr}_{m}f{g}^{{^{\prime}}{^{\prime}}}-{Pr}_{m}{f}^{{^{\prime}}{^{\prime}}}g=0,$$

11 $$\frac{{N}_{3}}{{N}_{4}}{\theta }^{{^{\prime}}{^{\prime}}}\left(\zeta \right)+Rd\frac{d}{d\zeta }\left({\left[1+ \theta \left(\zeta \right)\left({\theta }_{w}-1\right)\right]}^{3} {\theta }^{^{\prime}}\left(\zeta \right)\right)+Pr\left[f{\theta }^{^{\prime}}-{\Gamma }_{t}\left({f}^{2}{\theta }^{{^{\prime}}{^{\prime}}}+f{f}^{^{\prime}}{\theta }^{^{\prime}}\right)+\frac{1}{{N}_{4}}Q\theta \right]=0$$

The transformed non-dimensional associated boundary conditions are:

$$f=0, \; {f}^{^{\prime}}=1, \; g=0, \; {g}^{{^{\prime}}{^{\prime}}}=0, \; {N}_{3 }{\theta }^{^{\prime}}=Bi\left(\theta -1\right) \; \mathrm{ at } \; \zeta =0$$

12 $${f}^{^{\prime}}\to A, \; {g}^{^{\prime}}=1, \; \theta \to 0 \; \mathrm{ as } \; \zeta \to \infty ,$$

where $${N}_{1}=\frac{{\mu }_{thnf}}{{\mu }_{f}}, {N}_{2}= \frac{{\rho }_{thnf}}{{\rho }_{f}}, {N}_{3}=\frac{{k}_{thnf}}{{k}_{f}}, {N}_{4}=\frac{{(\rho {c}_{p})}_{thnf}}{{\left(\rho {c}_{p}\right)}_{f}}$$.

Here ${\phi }_{1},{\phi }_{2},$ and ${\phi }_{3}$ are the volume fractions of graphene oxide $\left(GO\right),$ gold $\left(Au\right)$ and Cobalt oxide $\left(C{o}_{3}{O}_{4}\right)$ nanoparticles respectively, ${\mu }_{tnf}$, ${\mu }_{f}$ are dynamic viscosities of ternary hybrid nanofluid and base fluid respectively, ${\sigma }_{tnf}$, ${\sigma }_{hnf}, {\sigma }_{f}$ are electrical conductivities of the ternary (tri-hybrid) nanofluid, hybrid nanofluid and base fluid respectively, ${k}_{tnf},{k}_{hnf}, {k}_{f}$ are thermal conductivity of tri-hybrid nanofluid, hybrid nanofluid and base fluid respectively, ${(\rho {c}_{p})}_{tnf},{(\rho {c}_{p})}_{f}$ are the specific heat capacitance of tri-hybrid nanofluid and base fluid respectively. The mathematical formulae for the thermo-physical characteristics of ternary hybrid nanofluid are described in Table 2.

**Table 2:** Mathematical relations of thermo-physical characteristics^47,55^.

Physical characteristics	Mathematical expressions
Density	$\frac{{\rho }_{thnf}}{{\rho }_{f}}=(1-{\phi }_{3})\left[\left(1-{\phi }_{2}\right)\left\{\left(1-{\phi }_{1}\right)+{\phi }_{1}\frac{{\rho }_{s1}}{{\rho }_{f}}\right\}+{\phi }_{2}\frac{{\rho }_{s2}}{{\rho }_{f}}\right]+{\phi }_{3}\frac{{\rho }_{s3}}{{\rho }_{f}}$
Dynamic viscosity	$\frac{{\mu }_{tnf}}{{\mu }_{f}}=\frac{1}{{(1-{\phi }_{1})}^{2.5}{(1-{\phi }_{2})}^{2.5}{(1-{\phi }_{3})}^{2.5}}$
Electrical conductivity	$\frac{{\sigma }_{tnf}}{{\sigma }_{hnf}}=\frac{{(1+{2\phi }_{3})\sigma }_{s3}+{(1-{2\phi }_{3})\sigma }_{hnf}}{{(1-{\phi }_{3})\sigma }_{s3}+{(1+{\phi }_{3})\sigma }_{hnf}},$ $\frac{{\sigma }_{hnf}}{{\sigma }_{nf}}=\frac{{\left(1+{2\phi }_{2}\right)\sigma }_{s2}+{\left(1-{2\phi }_{2}\right)\sigma }_{nf}}{{\left(1-{\phi }_{2}\right)\sigma }_{s2}+{\left(1+{\phi }_{2}\right)\sigma }_{nf}},$ $\frac{{\sigma }_{nf}}{{\sigma }_{f}}=\frac{{(1+{2\phi }_{1})\sigma }_{s1}+{(1-{2\phi }_{1})\sigma }_{f}}{{(1-{\phi }_{1})\sigma }_{s1}+{(1+{\phi }_{1})\sigma }_{f}}$
Thermal conductivity	$\frac{{k}_{tnf}}{{k}_{hnf}}=\frac{{k}_{s3}+2{k}_{hnf}-{2\phi }_{3}\left({k}_{hnf}-{k}_{s3}\right)}{{k}_{s3}+2{k}_{hnf}+{\phi }_{3}\left({k}_{hnf}-{k}_{s3}\right)},$ $\frac{{k}_{hnf}}{{k}_{f}}=\frac{{k}_{s2}+2{k}_{nf}-{2\phi }_{2}\left({k}_{nf}-{k}_{s2}\right)}{{k}_{s2}+2{k}_{nf}+{\phi }_{2}\left({k}_{nf}-{k}_{s2}\right)},$ $\frac{{k}_{nf}}{{k}_{f}}=\frac{{k}_{s1}+2{k}_{f}-{2\phi }_{1}\left({k}_{f}-{k}_{s1}\right)}{{k}_{s1}+2{k}_{f}+{\phi }_{1}\left({k}_{f}-{k}_{s1}\right)}$
Heat capacitance	$\frac{{(\rho {c}_{p})}_{tnf}}{{\left(\rho {c}_{p}\right)}_{f}}=\left(1-{\phi }_{3}\right)\left[\left(1-{\phi }_{2}\right)\left\{\left(1-{\phi }_{1}\right)+{\phi }_{1}\frac{{\left(\rho {c}_{p}\right)}_{s1}}{{\left(\rho {c}_{p}\right)}_{f}}\right\}+{\phi }_{2}\frac{{\left(\rho {c}_{p}\right)}_{s2}}{{\left(\rho {c}_{p}\right)}_{f}}\right]+{\phi }_{3}\frac{{\left(\rho {c}_{p}\right)}_{s3}}{{\left(\rho {c}_{p}\right)}_{f}}$

The wall characteristics such as the skin-friction coefficient and heat transfer rate (local Nusselt number) are important in materials processing systems. They are defined as:

13 $${C}_{f}={\left.\frac{{\tau }_{w}}{{\rho }_{f}{u}_{w}^{2}}\right|}_{y=0}$$

14 $$Nu={\left.\frac{x}{{k}_{f}\left({T}_{w}-{T}_{\infty }\right)}\left({q}_{r}-{k}_{thnf}\frac{\partial T}{\partial y}\right)\right|}_{y=0}$$

in which

15 $${\tau }_{w}={\mu }_{thnf}\left[\frac{\partial u}{\partial y}+\frac{\Gamma }{\sqrt{2}}{\left(\frac{\partial u}{\partial y}\right)}^{2}\right]$$

By introducing Eq. (8) in Eqs. (13) and (14), the following dimensionless expressions are obtained:

16 $${C}_{f}{Re}_{x}^\frac{1}{2}={N}_{1}f{^{\prime}}{^{\prime}}\left(0\right)\left[1+Wef{^{\prime}}{^{\prime}}\left(0\right)\right],$$

17 $$Nu{Re}_{x}^{-\frac{1}{2}}=\frac{1}{{N}_{4}}\left[1+Rd{N}_{4}{\left(1+ \theta \left(0\right)\left({\theta }_{w}-1\right)\right)}^{3}\right]\theta {^{\prime}}\left(0\right)$$

where ${Re}_{x}^\frac{1}{2}=x\sqrt{\frac{c}{{\vartheta }_{f}}}$ is the local Reynolds number.

In the present model, by constraining the volume fraction of $C{o}_{3}{O}_{4}$ nanoparticle to be zero i. e. ${\phi }_{3}=0$ then the model is reduced to the dual hybrid $GO-Au$ nanofluid model. When both volume fractions of $Au$ and $C{o}_{3}{O}_{4}$ nanoparticles are ignored i.e. ${\phi }_{2}={\phi }_{3}=0$ then the simplest case of the reduced $GO$ mono-nanofluid model is retrieved.

## Numerical solution procedure

The non-dimensional boundary layer Eqs. (9)–(11) with boundary conditions (12) are highly non-linear and coupled. Hence, it is very difficult to obtain exact solutions. Therefore, a numerical approach is adopted and the RK-4 method available in the bvp4c built-in function of MATLAB is utilized. By employing a numerical approach, the derivative of higher order equations is transformed into a system of first order equations, resulting in the following system^63^:

$${s}_{1}=f,$$

$${s}_{1}{^{\prime}}={s}_{2},$$

$${s}_{2}{^{\prime}}={s}_{3},$$

$${s}_{3}{^{\prime}}=-\frac{1}{\left(1+We{s}_{3}\right)}\left(\frac{{N}_{2}}{{N}_{1}}\left[f{f}^{{^{\prime}}{^{\prime}}}-{{f}^{^{\prime}}}^{2}+\left(\beta {{g}^{^{\prime}}}^{2}-g{g}^{{^{\prime}}{^{\prime}}}-1\right)\right]+{A}^{2}\right)$$

$${s}_{4}=g,$$

$${s}_{4}{^{\prime}}={s}_{5},$$

$${s}_{5}{^{\prime}}={s}_{6},$$

$${s}_{6}{^{\prime}}=-{Pr}_{m}\left[{s}_{1}{s}_{6}-{s}_{3}{s}_{4}\right],$$

$${s}_{7}=\theta ,$$

$${s}_{7}{^{\prime}}={s}_{8},$$

18 $$\left[\frac{{N}_{5}}{{N}_{4}}+Rd{\left[1+ {s}_{7}\left({\theta }_{w}-1\right)\right]}^{3}+Pr{\Gamma }_{t}{{s}_{1}}^{2}\right]{s}_{8}{^{\prime}}=-\left[3Rd{\left({\theta }_{w}-1\right)\left[1+{s}_{7}\left({\theta }_{w}-1\right)\right]}^{3}{{s}_{8}}^{2}+Pr\left({N}_{4}{s}_{1}{s}_{8}-{\Gamma }_{t}{{s}_{1}{s}_{2}s}_{8}+Q{s}_{7}\right)\right]$$

The relevant boundary conditions take the form:

$${s}_{1}=0, {s}_{2}=1, {s}_{4}=0, {s}_{8}{^{\prime}}=0, {N}_{5 }{s}_{8}=Bi\left({s}_{7}-1\right)\mathrm{ at }\zeta =0$$

19 $${s}_{2}\to A, {s}_{5}=1, {s}_{7}\to 0 \; \mathrm{ as } \; \zeta \to \infty$$

**Figure 2:**
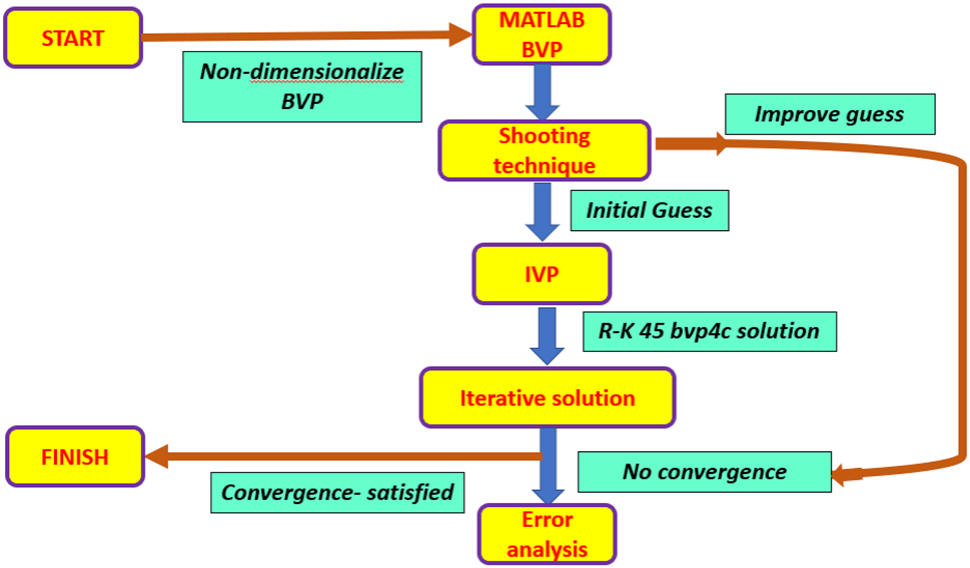
MATLAB bvp4c procedure.

The methodology is summarized in Fig. 2.

To assess the accuracy of the MATLAB bvp4c numerical solutions, benchmarking with earlier simpler models in the literature i.e. Hayat et al*.*^29^, Iqbal et al*.*^49^ and Animasaun et al*.*^63^ is conducted, for viscous fluids $({\phi }_{1}={\phi }_{2}={\phi }_{3}=0$), in the absence of radiation, heat source, non-Newtonian and non-Fourier effects ($Rd=Q= We={\Gamma }_{t}=0.0)$. The comparisons for the skin friction ${f}^{{^{\prime}}{^{\prime}}}\left(0\right)$ for different values of stretching parameter $A$ are revealed in Table 3. Significantly high agreement is observed between the solutions obtained using MATLAB and the findings reported in published works, confirming the accuracy of the present numerical methodology.

**Table 3:** Validation of present MATLAB bvp4c numerical solutions for ${f}^{{^{\prime}}{^{\prime}}}\left(0\right)$ with published studies when ${\phi }_{1}={\phi }_{2}={\phi }_{3}=0,Rd=Q= We={\Gamma }_{t}=0.0$.

$A$	Ref.^29^	Ref.^49^	Ref.^63^	Present MATLAB solutions
0.5	1.333012	1.333009	1.333016	1.3330115
1.0	1.006314	1.006318	1.006314	1.0063138
1.5	0.886717	0.886715	0.886717	0.8867164

## Graphical results and discussion

Comprehensive numerical results have been obtained via MATLAB bvp4c quadrature to evaluate the effect of designated thermal, magnetic, non-Newtonian and nanoscale parameters on transport characteristics in the ternary $GO-Au-C{o}_{3}{O}_{4}$ hybrid nanofluid coating boundary layer stagnation flow regime. Figures 3, 4, 5, 6, 7, 8, 9, 10, 11, 12, 13, 14, 15, 16, 17, 18, 19, 20, 21, 22, 23, 24, 25, 26, 27 and 28 visualize the profiles for velocity, induced magnetic field, temperature and streamline (iso-velocity) contours for all three ternary, hybrid and mono-nanofluid cases. The dimensionless parametric values are prescribed as follows (unless otherwise indicated): $\beta =0.2,We=0.3, {Pr}_{m}=0.1,Pr=4.1,$ $Q=0.2,Rd=0.5,{\theta }_{w}=1.2,{\Gamma }_{t}=0.1,A=0.5,{\phi }_{1}=0.01, {\phi }_{2}=0.02, {\phi }_{3}=0.03.$ All data has been extracted from the following references^77–83^ in addition to Refs.^47^ and ^57^ to represent as accurately as possible industrial smart nanocoating properties.

**Figure 3:**
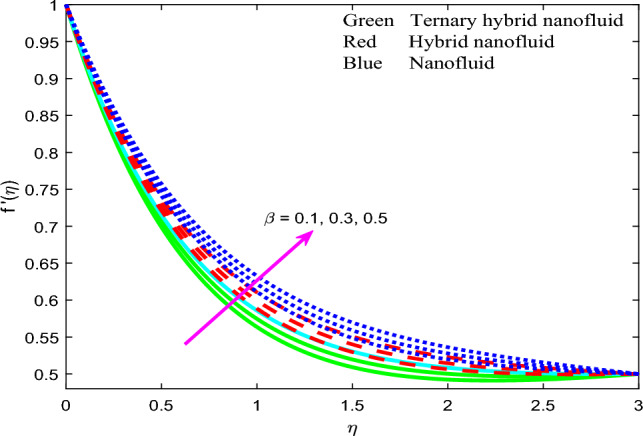
Profile of velocity $f{^{\prime}}\left(\zeta \right)$ for $\beta$.

**Figure 4:**
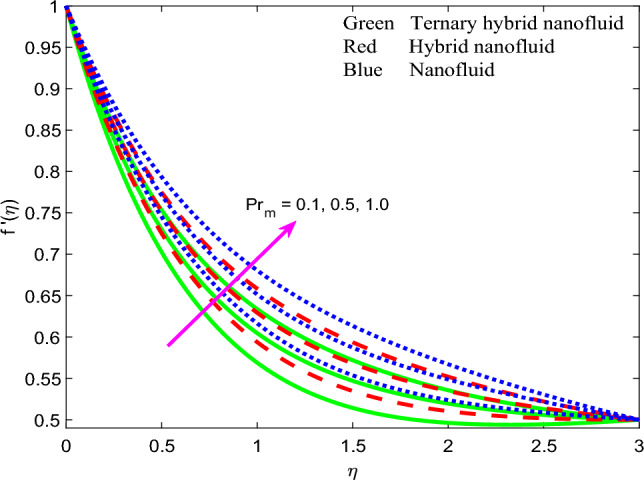
Profile of velocity $f{^{\prime}}\left(\zeta \right)$ for ${Pr}_{m}$.

**Figure 5:**
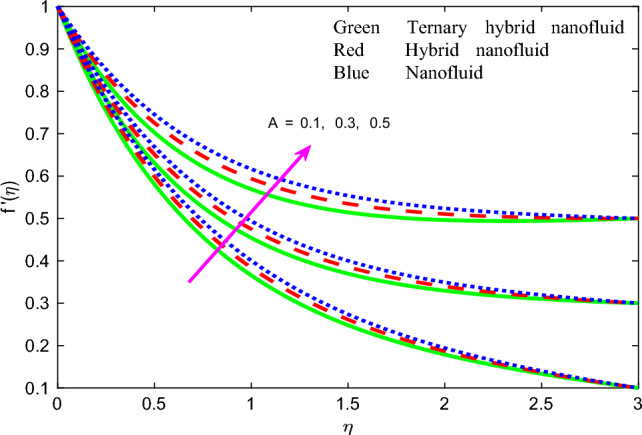
Profile of velocity $f{^{\prime}}\left(\zeta \right)$ for $A$.

**Figure 6:**
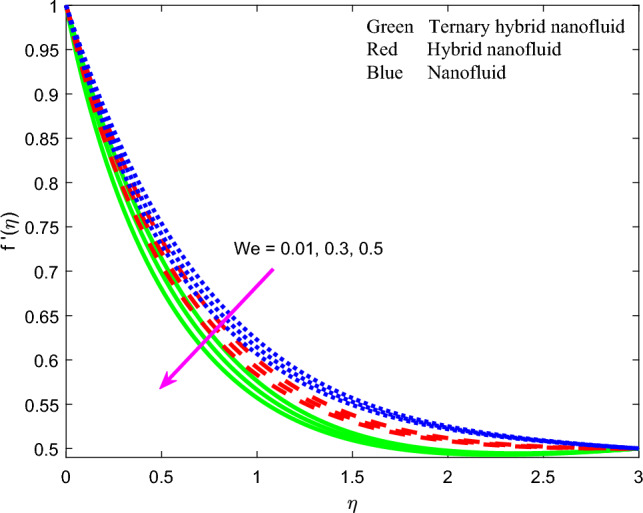
Profile of velocity $f{^{\prime}}\left(\zeta \right)$ for $We$.

**Figure 7:**
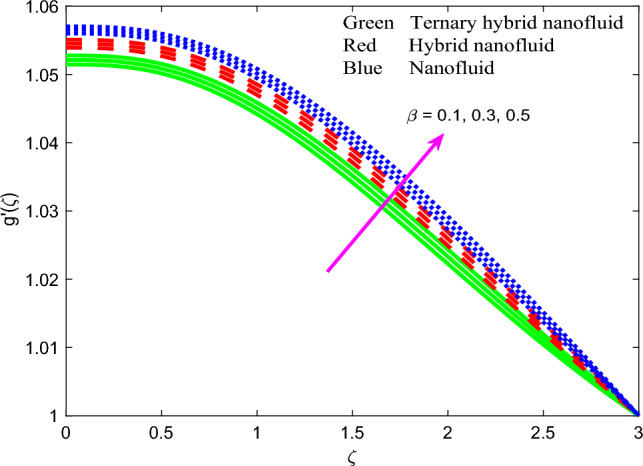
Profile of induced magnetic field $g{^{\prime}}\left(\zeta \right)$ for $\beta$.

**Figure 8:**
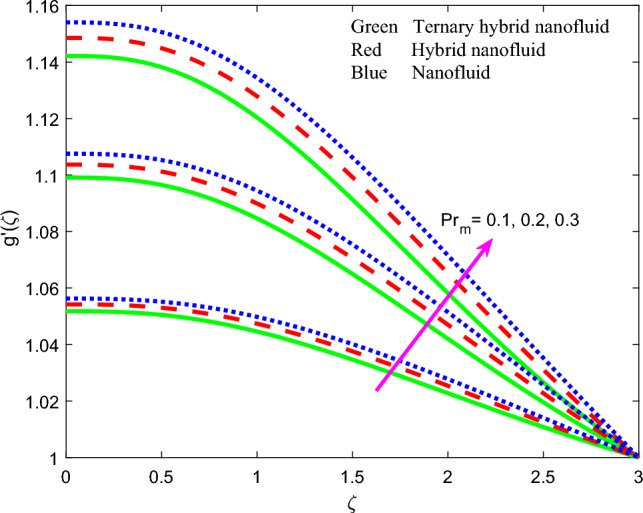
Profile of induced magnetic field $g{^{\prime}}\left(\zeta \right)$ for ${Pr}_{m}$.

**Figure 9:**
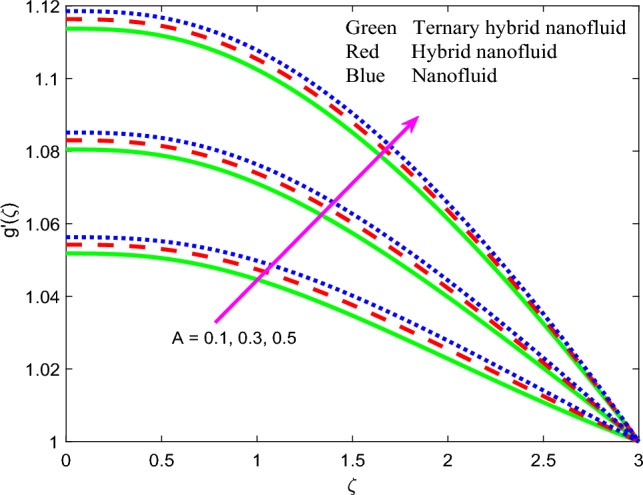
Profile of induced magnetic field $g{^{\prime}}\left(\zeta \right)$ for $A$.

**Figure 10:**
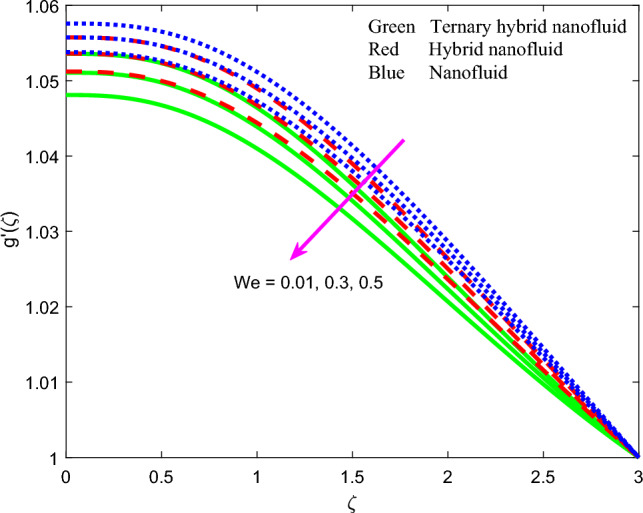
Profile of induced magnetic field $g{^{\prime}}\left(\zeta \right)$ for $We$.

**Figure 11:**
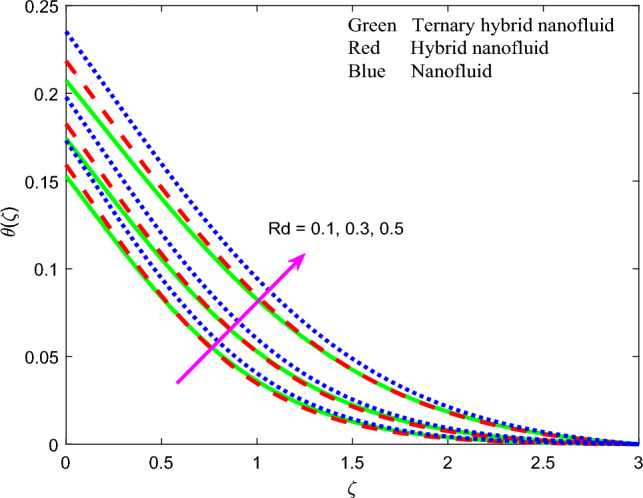
Profile of temperature $\theta \left(\zeta \right)$ for $Rd$.

**Figure 12:**
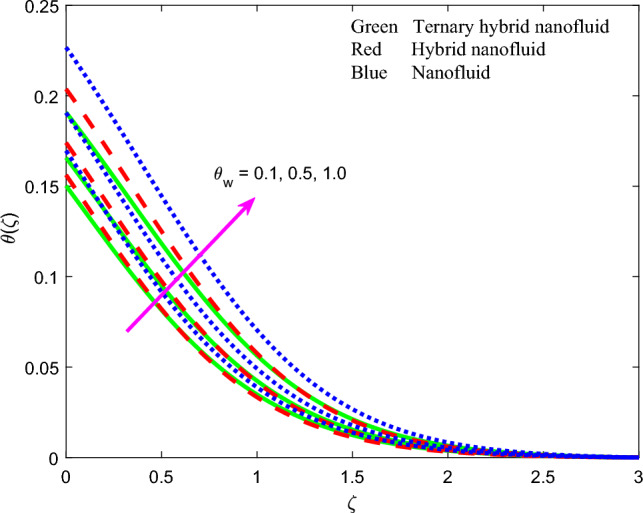
Profile of temperature $\theta \left(\zeta \right)$ for ${\theta }_{w}$.

**Figure 13:**
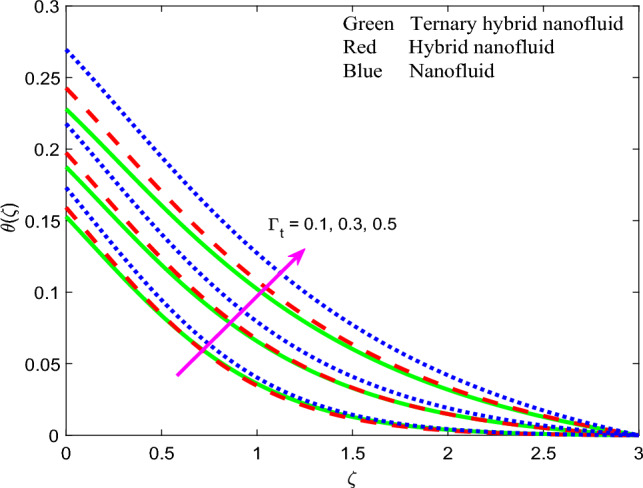
Profile of temperature $\theta \left(\zeta \right)$ for ${\Gamma }_{t}$.

**Figure 14:**
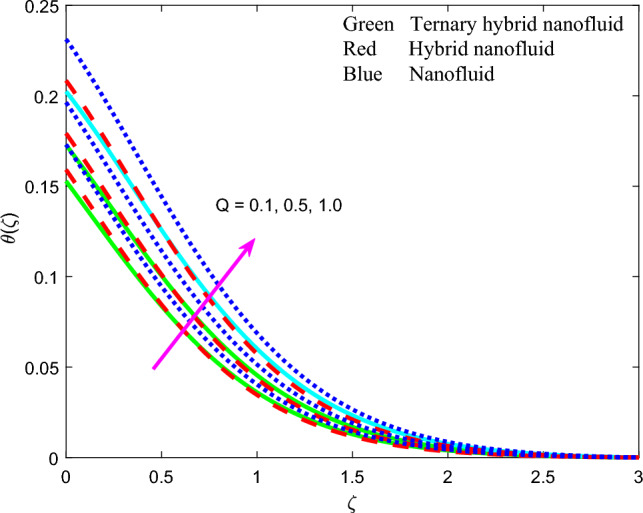
Profile of temperature $\theta \left(\zeta \right)$ for $Q$.

**Figure 15:**
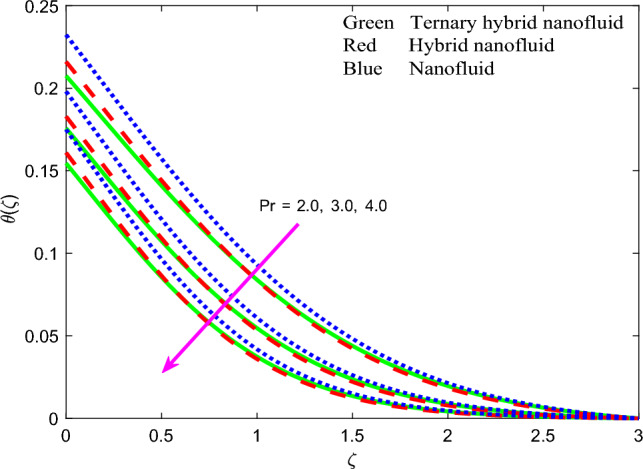
Profile of temperature $\theta \left(\zeta \right)$ for $Pr$.

**Figure 16:**
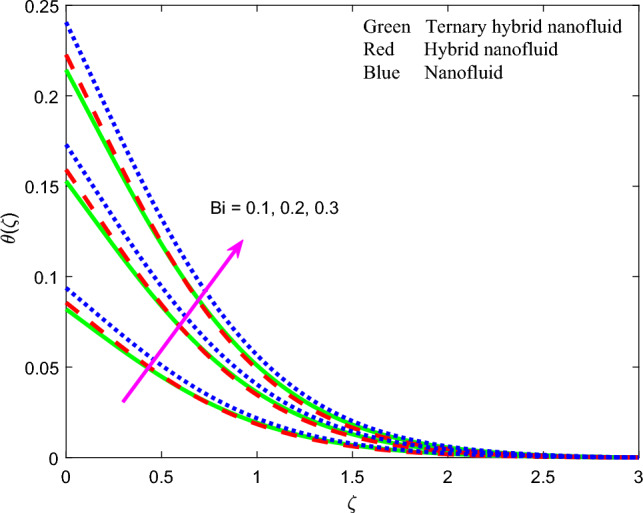
Profile of temperature $\theta \left(\zeta \right)$ for $Bi$.

**Figure 17:**
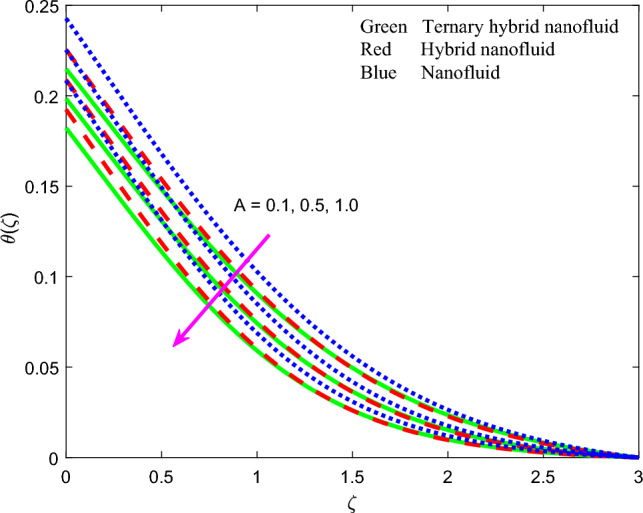
Profile of temperature $\theta \left(\zeta \right)$ for $A$.

**Figure 18:**
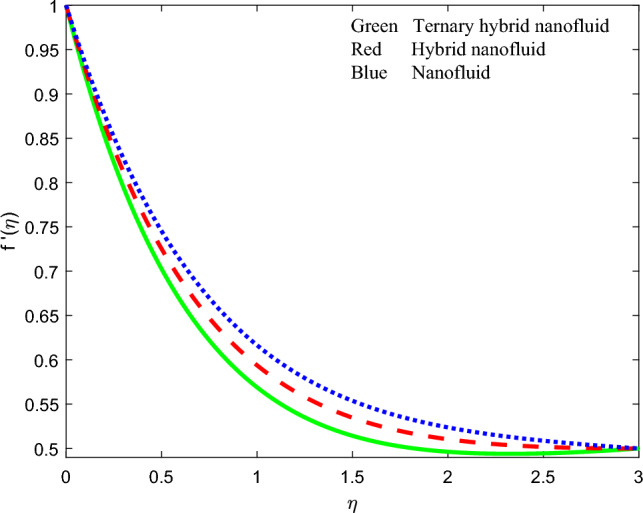
Comparison of velocity distributions $f{^{\prime}}\left(\zeta \right)$ for three nanofluids.

**Figure 19:**
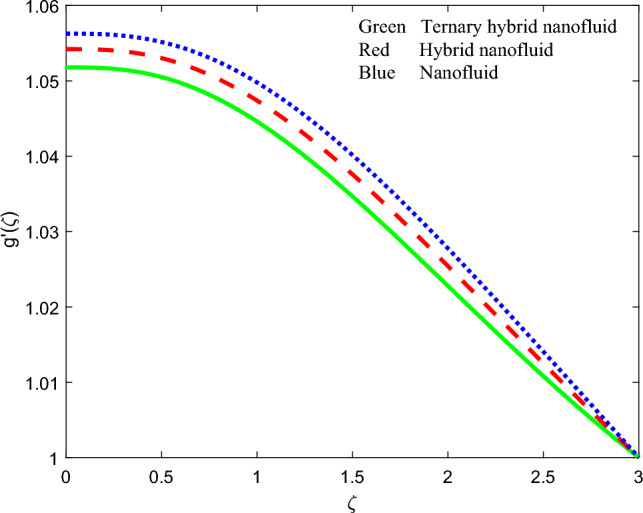
Comparison of induced magnetic field function profiles $g{^{\prime}}\left(\zeta \right)$ for three nanofluids.

**Figure 20:**
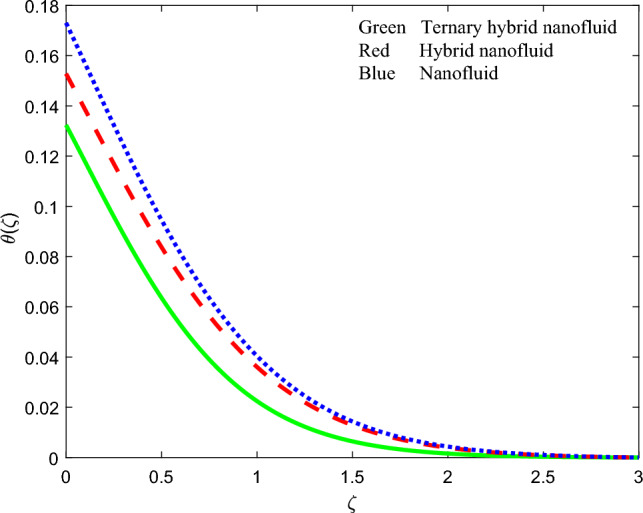
Comparison of temperature function $\theta \left(\zeta \right)$ for three nanofluids.

**Figure 21:**
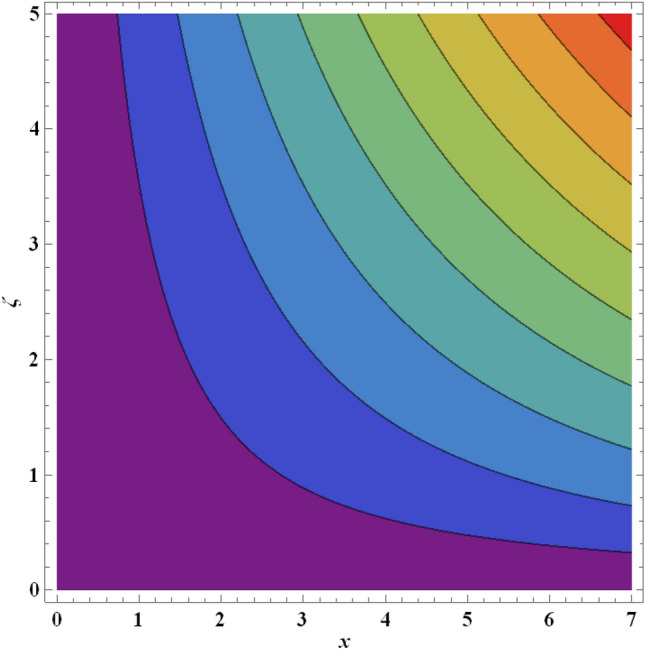
Streamlines for ternary nanofluid with $\beta =0.2$.

**Figure 22:**
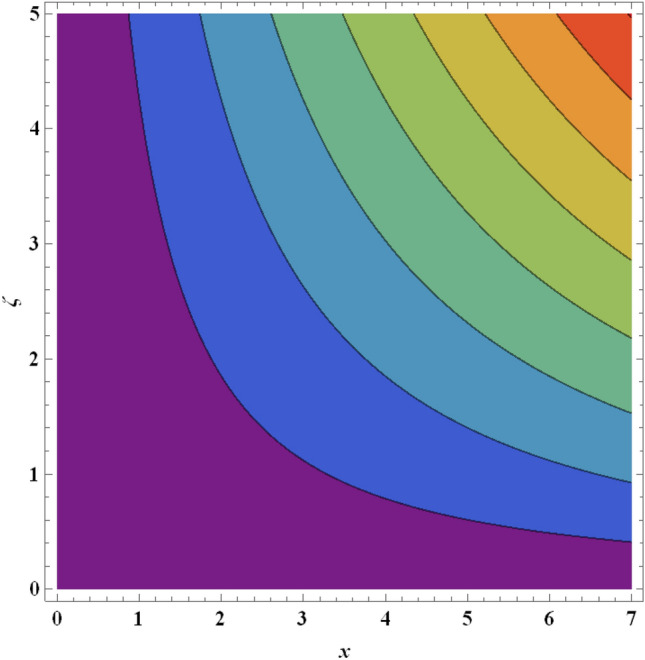
Streamlines for ternary nanofluid with $\beta =0.5$.

**Figure 23:**
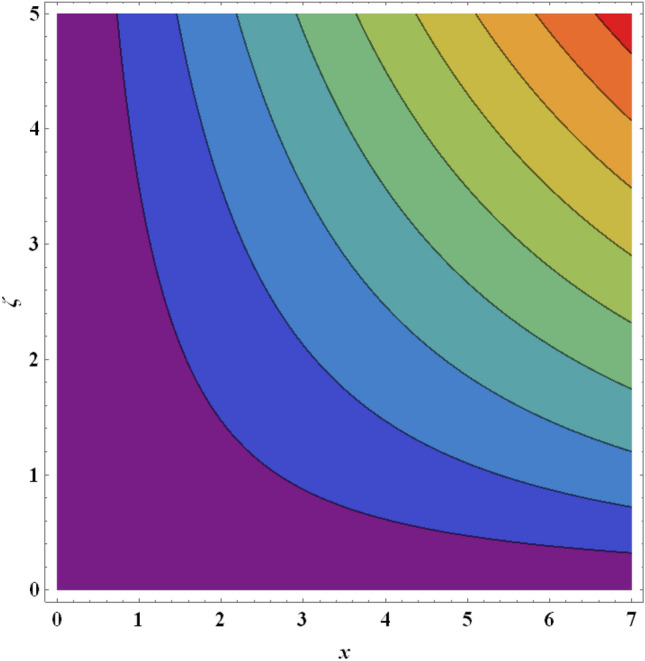
Streamlines for ternary nanofluid with $We=0.3$.

**Figure 24:**
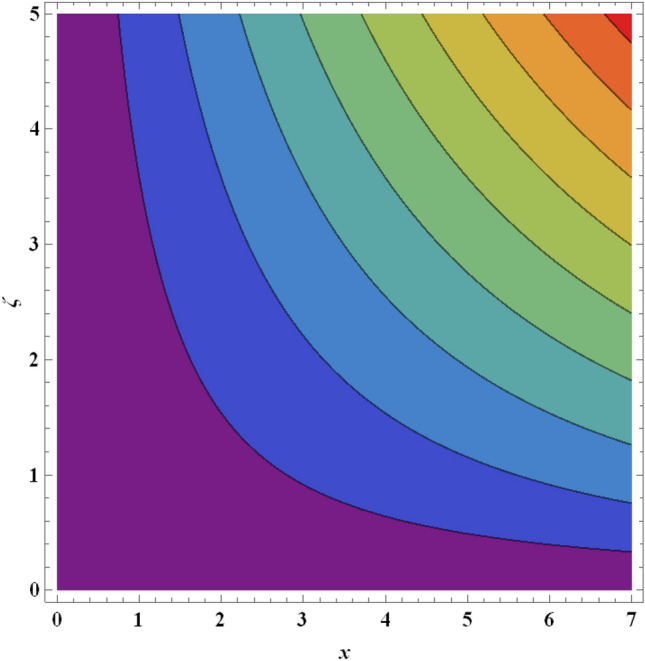
Streamlines for ternary nanofluid with $We=0.5$.

**Figure 25:**
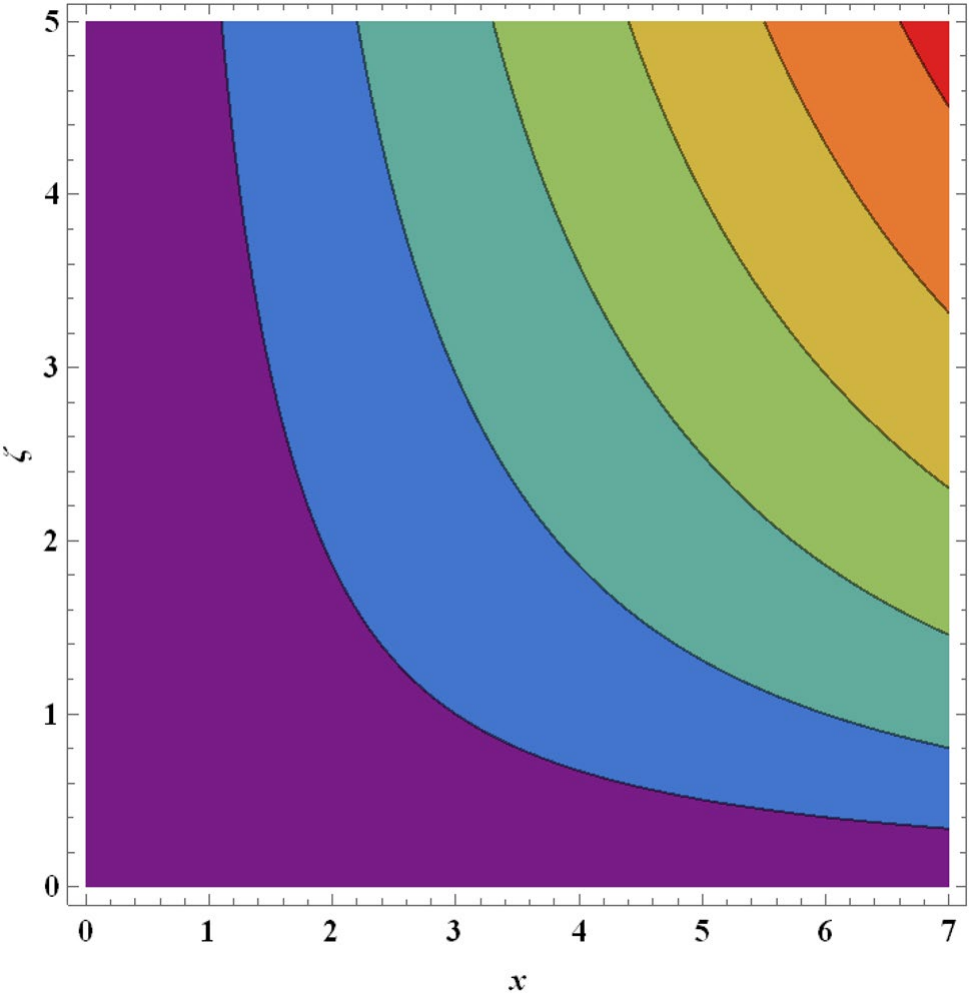
Streamlines for ternary nanofluid with $A=0.2$.

**Figure 26:**
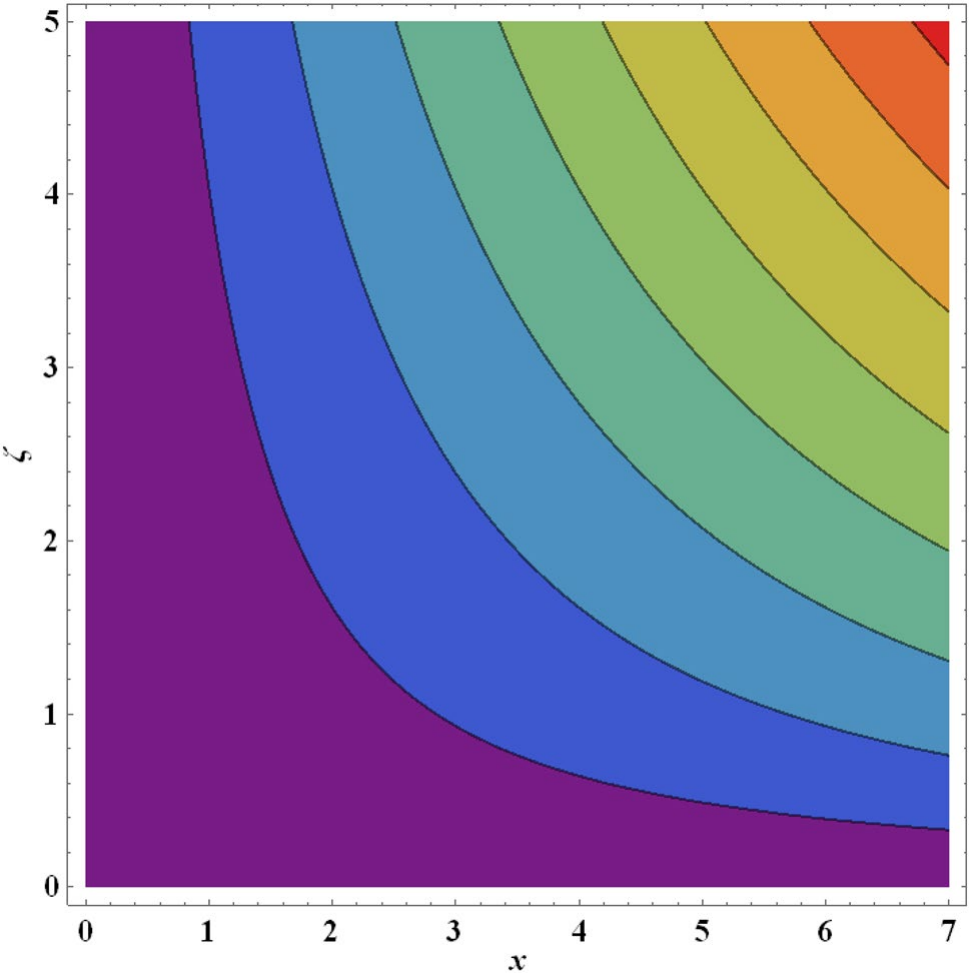
Streamlines for ternary nanofluid with $A=0.4$.

**Figure 27:**
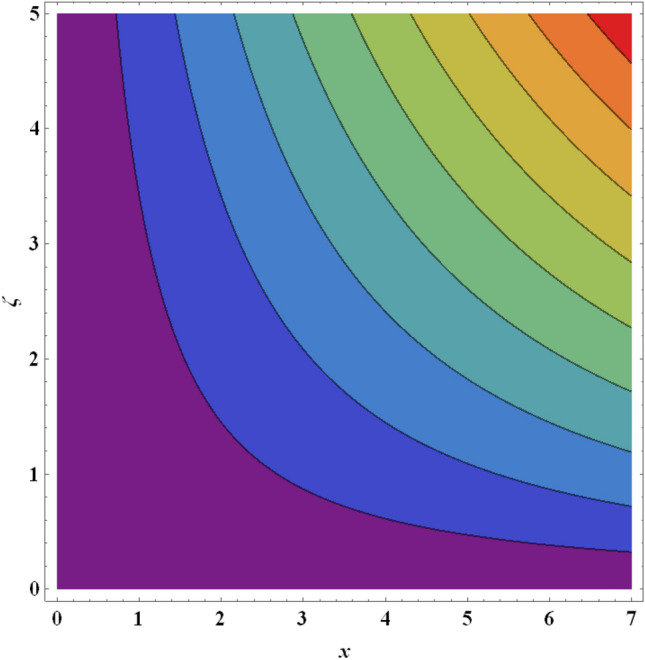
Streamlines for ternary nanofluid with ${Pr}_{m}=0.2$.

**Figure 28:**
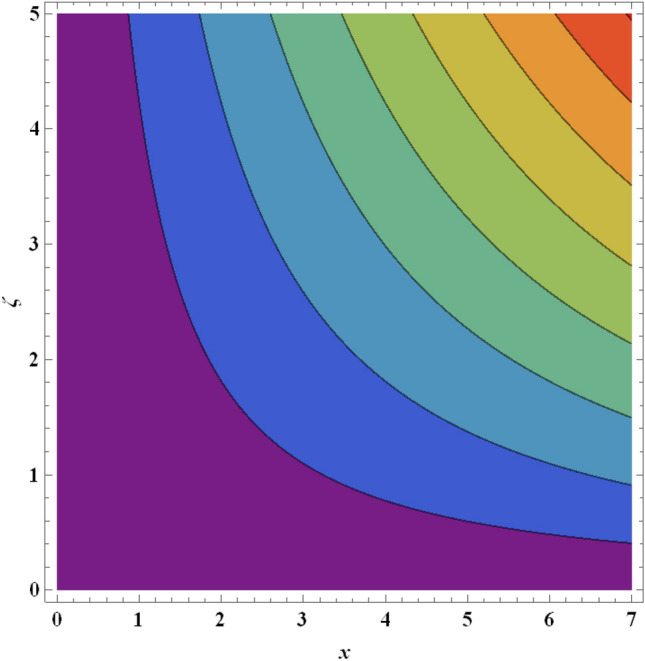
Streamlines for ternary nanofluid with ${Pr}_{m}=0.5$.

### Velocity field $\left(f{^{\prime}}\left(\zeta \right)\right)$

The velocity function $f{^{\prime}}\left(\zeta \right)$ versus transverse coordinate (*η*) for the influence of magnetic parameter $\beta$, magnetic Prandtl number ${Pr}_{m}$, stretching parameter $A$ and Weissenberg number $We$ for all three nanofluid cases are depicted in Figs. 3, 4, 5 and 6. The response in velocity $f{^{\prime}}\left(\zeta \right)$ to a variation in magnetic parameter, $\beta$ is illustrated in Fig. 3. The parameter $\beta =\frac{{\mu }_{e}{H}_{0}^{2}}{4\pi {\rho }_{f}{c}^{2}}$ and is directly proportional to the applied magnetic field, *H*_*o*_. It features in the quadratic term in the reduced momentum Eq. (9), $+\frac{{N}_{2}}{{N}_{1}}\left[+\left(\beta {{g}^{^{\prime}}}^{2}-g{g}^{{^{\prime}}{^{\prime}}}-1\right)\right]$ which couples the induced magnetic field to the velocity field. This term is clearly assistive and aids in momentum development resulting in a boost in the velocity magnitudes and a reduction in hydrodynamic boundary layer thickness. For all $\beta$ values, the computation reveals asymptotically smooth decays in the velocity field spanning from the wall to the free stream, thus affirming the implementation of a suitably large infinity boundary condition in the MATLAB code.

Significant flow deceleration is observed for the ternary (tri-hybrid) $GO$–$Au$–$C{o}_{3}{O}_{4}$ engine oil-nanofluid whereas strong flow acceleration is computed for the cobalt oxide $\left(C{o}_{3}{O}_{4}\right)$-monofluid at all values of magnetic parameter $\beta$. The dual hybrid nanofluid case ($GO-Au$) achieves intermediate velocity magnitudes between the other two cases. The boost in viscosity in the ternary nanofluid case is the most dramatic. This produces greater resistance to the bulk nanofluid transport in the boundary layer and leads to deceleration. However, boundary layer flow reversal or separation is never induced in the regime as confirmed by consistently positive values of velocity at all locations transverse to the wall. Enhanced flow control during the coating process can therefore best be achieved with the ternary hybrid nanofluid.

The influence of magnetic Prandtl number ${Pr}_{m}$ $\left({Pr}_{m} = 0.1, 0.5, 1.0\right)$ on dimensionless velocity profiles is illustrated in Fig. 4. There is a substantial elevation in velocity field with increment in magnetic Prandtl number ${Pr}_{m}$. Again, velocity is highest for unitary nonfluid as contrasted to that of two other nanofluids (dual hybrid and ternary hybrid) and again this is attributable to the lower viscosity of the mono-nanofluid. This produces a weaker resistance to the boundary layer flow which enables improved acceleration. ${Pr}_{m}=\frac{{\vartheta }_{f}}{{\alpha }_{m}}$ and embodies the ratio of magnetic diffusion rate to viscous diffusion rate. Additionally, it represents the ratio between the magnetic Reynolds number and the ordinary Reynolds number. This embodiment is evident in the second-order terms present in the magnetic induction conservation Eq. (10) viz, $+{Pr}_{m}f{g}^{{^{\prime}}{^{\prime}}}$ and $-{Pr}_{m}{f}^{{^{\prime}}{^{\prime}}}g$. It is noteworthy that the applied magnetic field *H*_*0*_ acts perpendicularly to the surface (in the y-direction). The induced magnetic field *H*_*2*_'s normal component diminishes at the wall, while the parallel component *H*_*1*_ approaches the value of *H*_*0*_. Additionally, due to the assumption of finite conductivity in the nanofluid and the non-conductivity of the wall (sheet), there is no presence of a surface current sheet. This results in the continuity of the tangential component of the magnetic field across the interface. Since polarization voltage is also zero at the wall, no energy is added or extracted from the nanofluid via electrical means. When *Pr*_*m*_ = *1* both the viscous and magnetic diffusion rate are equivalent, and the corresponding hydrodynamic and magnetic boundary layers have equal thickness. This case produces maximum intensity in the induced magnetic stream function gradient which in turn accelerates the flow leading to a minimal velocity boundary layer thickness. Conversely when *Pr*_*m*_ < *1, the viscous diffusion rate is swamped by magnetic diffusivity* which produces minimal velocity magnitudes. A thicker momentum boundary layer is produced in the coating. Similar observations have been reported in Ali et al.^26^ and Iqbal et al*.*^49^ and also much earlier for Newtonian conducting fluids by Hughes and Young^84^. Clearly there is an intricate interplay between the magnetic induction and velocity fields which justifies the inclusion of magnetic induction in smart coating mathematical models. Figure 5 visualizes the impact of stretching parameter $A$ on velocity field for three nanofluids. It is noticed that the velocity is escalating function of $A$ since supplementary momentum is impulsively transferred to the boundary layer via linear wall stretching. Hydrodynamic boundary layer thickness is therefore depleted with accelerated shearing of the nanofluid along the wall. In contrast to classical boundary layer flow, the wall velocity is not vanishing and is in fact maximized. The free stream velocity is minimized in the present scenario. Weak stretching (*A* = 0.1) naturally produces lower velocities than strong stretching (*A* = 0.5). Again *mono-nanofluid* produces markedly greater velocity magnitudes than the other two hybrid nanofluids. The impact of Weissenberg number $We$ is highlighted on the nanofluid velocity in Fig. 6. For all three nanofluids, there is a consistent depletion in velocity with increasing $We$. The Weissenberg number $We$ is required to simulate the nonlinear relation between shear stress and strain rate in the non-Newtonian nanofluid. It characterizes the ratio of elastic to viscous forces in the nanofluid. It also expresses the ratio of fluid relaxation time to specific time. For large values of *We*, fluid relaxation time will greatly exceed the time scale of the flow and elastic stresses will be dominant. The reverse behaviour will arise when relaxation time is exceeded by time sale of the flow for which the viscous effects will dominate, and elastic effects will subside. The nanofluid is therefore able to move with less tensile stress impedance when *We* is reduced and this produces the observed acceleration and increment in velocities. Hydrodynamic boundary layer thickness is therefore reduced with smaller *We* values whereas it is increased (flow deceleration) with higher We values. The nano-coating homogeneity and thickness consistency are in due course strongly influenced by the Weissenberg number, as emphasized by Vidales-Herrera and López^84^. As before the mono-nanofluid produces highest velocity magnitudes relative to the two hybrid nanofluids.

### Induced magnetic field $\left(g{^{\prime}}\left(\zeta \right)\right)$

The effect of magnetic parameter $\beta$, magnetic Prandtl number ${Pr}_{m}$, stretching rate parameter $A$ and Weissenberg number $We$ on induced magnetic field stream function gradient profiles, $g{^{\prime}}\left(\zeta \right)$ is reported in Figs. 7, 8, 9 and 10 for all three nanofluids. Figure 7 shows the response in $g{^{\prime}}\left(\zeta \right)$ to different values of magnetic parameter $\beta$. $g{^{\prime}}\left(\zeta \right)$ is clearly an increasing function of magnetic variable $\beta$. It is noteworthy that the magnetic parameter, $\beta =\frac{{\mu }_{e}{H}_{0}^{2}}{4\pi {\rho }_{f}{c}^{2}}$, also expresses the ratio of the Alfven speed to the free stream velocity. For realistic simulations, this parameter has to be constrained in the range 0 ≤ $\beta$  ≤ 1 to maintain consistency with the steady-state solution of the so-called *Super-Alfven* flow in MHD coating systems. This guarantees that the Alfven wave speed is exceeded by the free stream velocity. When $\beta$ > 1 *sub-Alfven flow* arises in which any disturbance within the boundary layer regime can propagate upstream via magnetohydrodynamic Alfven waves, and this scenario is not relevant to the present study^84^. Since the applied magnetic field (*H*_*o*_) appears as a quadratic function in the definition of $\beta$, stronger magnetic field intensity will dramatically boost the values of $\beta$. In turn this will elevate the magnetic field stream function gradient and produce a much thicker magnetic boundary layer on the stretching wall. The profiles are inverse parabolic in nature. Ternary nanofluid generates strong damping in the magnetic induction field whereas mono-nanofluid accentuates it. This response is connected to the very different electrical conductivities of the nanofluids considered which in turn are modified by the individual contributions of the different nanoparticles. In the mono-nanofluid case only one nanoparticle contributes to this effect. Significant control of the magnetic induction is therefore optimized with the ternary nanofluid. Figure 8 shows that a boost in magnetic Prandtl number ${Pr}_{m}$ also enhances the induced magnetic stream field function gradient $g{^{\prime}}\left(\zeta \right)$. As elaborated earlier, the magnetic diffusivity is lower with greater values of magnetic Prandtl number. This results in a damping in the magnetic induction field. $g{^{\prime}}\left(\zeta \right)$ values are lowest for the ternary $GO$–$Au$–$C{o}_{3}{O}_{4}$ hybrid nanofluid whereas it is highest for the $GO$ unitary nanofluid case. This may be attributable to the combined presence of magnetic and carbon-based nanoparticles in the former and the sole presence of only carbon-based nanoparticles (graphene oxide) in the latter. These nanomaterials have very different electrical conductivities which influences the effect on the induced magnetic field. The dual $GO-Au$ hybrid nanofluid produces $g{^{\prime}}\left(\zeta \right)$ values in between the mono and ternary nanofluids which is probably attributable to the balance in volume fraction of both carbon (graphene oxide) and metallic (gold) nanoparticles. Figure 9 shows that induced magnetic stream field function gradient $g{^{\prime}}\left(\zeta \right)$ is boosted with higher values of stretching parameter $A$. While there is no direct contribution of sheet stretching in the magnetic induction Eq. (10), the parameter does arise in the tern, $+{A}^{2}$ in the momentum Eq. (9) which also features coupling terms, $\left[+\left(\beta {{g}^{^{\prime}}}^{2}-g{g}^{{^{\prime}}{^{\prime}}}\right)\right]$. The stretching parameter therefore indirectly influences the magnetic induction field via the coupling with the velocity field in Eq. (9) and additionally via the boundary condition, $f{^{\prime}}\to A$ in Eq. (12). Flow acceleration with greater A values therefore also boosts the induced magnetic field values. Again, the mono-nanofluid (GO) achieves the maximum magnetic induction magnitudes relative to the two hybrid nanofluids. Figure 10 shows that a strong depletion in magnetic stream function gradient, with increasing Weissenberg number $We$, which is associated intimately with the deceleration induced (as shown earlier in Fig. 6. The magnetic boundary layer thickness therefore diminishes with increasing Weissenberg number (weaker elastic effect). Ternary hybrid nanofluid produces the lowest magnitudes of magnetic induction (thinnest magnetic boundary layer) whereas the mono-nanofluid attains the highest magnitudes (thickest magnetic boundary layer). Clearly therefore even the rheology of the nanofluid exerts a prominent effect on the magnetic induction distribution in the coating regime. The physical insights can only be captured by including a robust non-Newtonian model and this is not achievable with classical Navier–Stokes formulations.

### ***Temperature field***$\left(\theta \left(\zeta \right)\right)$

The influence of thermal radiation parameter $$Rd$$, temperature ratio parameter, $${\theta }_{w}$$, non-Fourier thermal relaxation time $${\Gamma }_{t}$$, heat source parameter $$Q$$, Prandtl number $$Pr$$, Biot number $$Bi$$ and wall stretching parameter $$A$$ on temperature profiles, $$\theta \left(\zeta \right)$$ is visualized in Figs. 11, 12, 13, 14, 15, 16 and 17 again for all three nanofluid types. Figure 11 indicates that when $$Rd$$ is increased, the temperature of all three nanofluids is sizeably enhanced. $$Rd= \frac{16{\sigma }^{*}{T}_{\infty }^{3}}{3{k}^{*}{k}_{f}}$$ and features in the augmented thermal diffusion term in the energy (thermal boundary layer) Eq. (11), viz, $$Rd\frac{d}{d\zeta }\left({\left[1+ \theta \left(\zeta \right)\left({\theta }_{w}-1\right)\right]}^{3} {\theta }^{^{\prime}}\left(\zeta \right)\right)$$. When radiation heat transfer is absent this term is negated. $$Rd$$ is also known as the Boltzmann number. When *Rd* = 1 both heat transfer modes contribute equally. When *Rd* > 1 thermal radiation dominates and vice versa for *Rd* < 1. Significant energization of the nanofluid is produced with increment in radiative flux even when *Rd* < 1, as observed in Fig. 10. This exacerbates thermal diffusion and also molecular conduction which leads to a thicker thermal boundary layer. A more dramatic modification in temperatures is induced at the wall and progressively diminished through the boundary layer. Ternary nanofluid curtails however the heating effect whereas mono-nanofluid accentuates it. Figure 12 shows that an elevation in temperature ratio variable $${\theta }_{w}$$ strongly enhances temperature magnitudes. $${\theta }_{w}=\frac{{T}_{w}}{{T}_{\infty }},$$ and expresses the ratio of the wall temperature to the free stream temperature. When this parameter is unity both temperatures are identical. In Fig. 12, all the profiles correspond to $${\theta }_{w}$$ < 1, for which the free stream temperature exceeds the wall temperature. This will strongly influence heat transfer in the boundary layer via thermal convection currents. A boost in $${\theta }_{w}$$ therefore boosts thermal diffusion in the boundary layer, increases temperatures and also thermal boundary layer thickness. However again the mono-nanofluid produces more impressive temperatures relative to the other two hybrid nanofluids. The effects of thermal relaxation time variable $${\Gamma }_{t}$$ ($$=0.1, 0.3, 0.5)$$ on fluid temperature is displayed in Fig. 13. It is perceived that with amplification of $${\Gamma }_{t}$$, the temperatures are strongly increased at all values of transverse coordinate, *η.* The non-Fourier model substantially modifies the heat flux terms and introduces hyperbolic finite wave conduction effects known as thermal relaxation. These feature in the terms, $$-{\Gamma }_{t}\left({f}^{2}{\theta }^{{^{\prime}}{^{\prime}}}+f{f}^{^{\prime}}{\theta }^{^{\prime}}\right)$$ in Eq. (11). The classical Fourier model negates this effect and is a parabolic model. With increasing $${\Gamma }_{t}$$ values the heat flux in all three nanofluids is elevated. This generates an intensification in thermal conduction between the nanoparticles which increases thermal boundary layer thickness. The Fourier model will under-predict the temperature distribution and also thermal boundary layer thickness. Figure 14 reveals that with an increment in heat source parameter $$Q$$*, temperature* $$\theta \left(\zeta \right)$$ is considerably enhanced. In materials processing a hot spot may be embedded on the substrate to achieve this boost in volumetric heat generation. Thermal boundary layer thickness will also be elevated. In both Figs. 13 and 14, the mono-nanofluid (graphene $$GO$$/$$EO$$) invariably produces higher temperatures than the other two hybrid nano fluids. Figure 15 demonstrates that with an increment in Prandtl number $$Pr$$ there is a marked depression in temperature $$\theta \left(\zeta \right)$$ which is observed for all three nanofluids. A strong depletion is also generated in thermal boundary layer thickness. Higher Prandtl numbers (*Pr* > 1, corresponding to metallic-nanoparticle oil nanofluids) imply a much lower thermal conductivity of the nano-polymer. This circumvents thermal diffusion and cools the regime. For *Pr* = *1* both thermal diffusion and momentum diffusion rates are equivalent, and temperature is a maximum as is thermal boundary layer thickness. The thermal diffusivity of the nanofluid is controlled also by the nanoparticle thermal conductivity. Careful selection and volume fractions of specific nanoparticles can therefore enable excellent manipulation of the heat transfer characteristics in magnetic nano-coatings^80,81^. As noted in earlier plots, ternary hybrid nanofluid exhibits lowest temperatures. It is apparent from Fig. 16 that the fluid temperature is enhanced with elevation in the thermal Biot number $$Bi$$. This parameter is defined as $$Bi=\sqrt{\frac{{\vartheta }_{f}}{c}} \frac{{h}_{f}}{{k}_{f}}$$. It represents the ratio of the thermal resistance for conduction inside the boundary layer to the resistance for convection at the surface (wall). It arises only in the augmented convective wall thermal boundary condition (12), $${N}_{3 }{\theta }^{^{\prime}}=Bi\left(\theta -1\right)\mathrm{ at }\zeta =0$$. Values of thermal Biot number lower than 0.1 indicate that the rate of thermal conduction within the body surpasses the heat convection away from its surface. Consequently, the temperature gradients occurring internally can be disregarded. This temperature range is unsuitable for materials processing operations as a Biot number below 0.1 indicates "thermally thin" scenarios. In this particular investigation, the Biot number is maintained at a minimum of 0.1 or higher, which corresponds to "thermally-thick" regimes. The Biot number exhibits a direct relationship with the convection heat transfer coefficient at the wall and an inverse relationship with thermal conductivity. Overall, the increase in Biot number improves the intensity of convective heating of the surface and this encourages thermal diffusion leading to a boost in temperature and thermal boundary layer thickness. The most dramatic elevation in temperature is computed at the wall. Ternary and dual hybrid nanofluids achieve approximately the same temperatures whereas as the graphene oxide mono-nanofluid produces much higher values. The temperature $$\theta \left(\zeta \right)$$ as depicted in Fig. 17 is found to decline considerably with larger values of the stretching parameter, $$A$$. The acceleration produced in the stretching regime on either side of the stagnation point, implies that momentum diffusion is boosted. This will exceed the thermal diffusion and will result in inhibited convection in the boundary layer. Thermal boundary layer thickness will therefore be reduced. $$GO$$ Mono-nanofluid generates maximum temperatures in comparison with $$GO$$- $$Au$$ dual and $$GO$$–$$Au$$–$$C{o}_{3}{O}_{4}$$ ternary hybrid nanofluid.

### Comparison of streamline contours for the three nanofluids

To summarize the relative performance of ternary, hybrid and unitary nanofluids, the velocity profile, induced magnetic stream function gradient and temperature profiles are displayed in Figs. 18, 19 and 20 accordingly. From these plots, the tri-hybrid $GO-Au-C{o}_{3}{O}_{4}$ nanofluid clearly produces minimal velocity, induced magnetic field and temperature magnitudes whereas the mono graphene nanofluid achieves the highest magnitudes. The dual $GO-Au$ hybrid nanofluid results fall in between these other two nanofluids.

### Streamlines

The streamline contour distributions in the regime for different values of magnetic parameter $\beta$, Weissenberg number $We$, stretching parameter $A$ and magnetic Prandtl number ${Pr}_{m}$ for the case of ternary $GO-Au-C{o}_{3}{O}_{4}$ hybrid nanofluid are illustrated in Figs. 21, 22, 23, 24, 25, 26, 27 and 28. Comparing Figs. 21 and 22, there is observed to be a growth in the higher magnitude streamlines in the top right corner (red zone) as magnetic parameter $\beta$ increases from 0.2 to 0.5. The streamline pattern is therefore clearly modified by magnetic field intensity. A large deceleration zone is however maintained in the lower left, upper left and lower right corners. With an increment in Weissenberg number $We$ from 0.3 to 0.5 (Figs. 23, 24), however the top right high intensity flow zone is contracted indicating deceleration is induced in this region. An increment in Weissenberg number $We$ alters the relative contribution of elastic and viscous forces in the regime which modifies the flow pattern in the boundary layer. Figures 25 and 26 that the greater values of stretching parameter $A$, also primarily alter the streamline magnitudes in the upper right corner. Figures 27 and 28 indicate that a significant deceleration in the flow is produced again in the upper right corner zone with an increment in magnetic Prandtl number ${Pr}_{m}$ from 0.2 to 0.5. All parameters studied therefore clearly exhibit a tangible influence on the streamline distributions.

### Skin-friction coefficient and Nusselt number

Table 4 displays the influence of $\beta , We, A$ and ${Pr}_{m}$ on skin friction, $-{Re}_{x}^\frac{1}{2}{C}_{f}$ for all three nanofluids. Evidently the skin friction (dimensionless surface shear stress at the wall) is reduced with increment in magnetic parameter $\beta$, Weissenberg number $We$, stretching parameter $A$ and magnetic Prandtl number ${Pr}_{m}$. Much higher skin friction magnitudes are computed for ternary hybrid $GO$–$Au$–$C{o}_{3}{O}_{4}/EO$ nanofluid as comparted to the other two nanofluids. The impact of ${\Gamma }_{t}$, $Rd, {\theta }_{w}, Pr, Q$ and $Bi$ on local Nusselt number, $-{Re}_{x}^{-\frac{1}{2}}Nu$ is elucidated in Table 5. The rate of heat transfer *to the wall* is enhanced with increment in thermal radiation, temperature ratio, heat source and Prandtl number while the reverse trend is observed for larger values of thermal relaxation time (non-Fourier parameter) and Biot number. Furthermore, the rate of heat transfer for the unitary nanofluid $GO/EO$ is lowest in comparison with the hybrid binary ($GO$–$Au/EO$) and ternary $GO$–$Au$–$C{o}_{3}{O}_{4}/EO$ nanofluids.

**Table 4:** Skin friction, $-{Re}_{x}^\frac{1}{2}{C}_{f}$, for the three distinct nanofluids with different parameters.

$\beta$	$We$	$A$	${Pr}_{m}$	$-{Re}_{x}^\frac{1}{2}{C}_{f}$		
				Ternary hybrid nanofluid	Dual hybrid nanofluid	Mono-nanofluid
0.1				0.878489	0.743947	0.648585
0.2				0.867413	0.733943	0.639361
0.3				0.856184	0.723821	0.630043
	0.1			0.895019	0.754943	0.655982
	0.2			0.881685	0.744756	0.647891
	0.3			0.867413	0.733943	0.639361
		0.1		1.132955	0.992731	0.895887
		0.3		1.032046	0.894244	0.798170
		0.5		0.867413	0.733943	0.639361
			0.2	0.846543	0.715051	0.621905
			0.5	0.791773	0.665467	0.576064
			0.7	0.761752	0.638328	0.550989

**Table 5:** Nusselt number$,-{Re}_{x}^{-\frac{1}{2}}Nu$ for the three distinct nanofluids with different parameters.

${\Gamma }_{t}$	Rd	${\theta }_{w}$	$Pr$	$Q$	$Bi$	$-{Re}_{x}^{-\frac{1}{2}}Nu$		
						Ternary hybrid nanofluid	Dual hybrid nanofluid	Mono-nanofluid
0.2						0.309736	0.306797	0.300474
0.5						0.287818	0.282341	0.272324
0.7						0.272024	0.264621	0.252622
	1					0.450565	0.446013	0.437684
	2					0.706555	0.696580	0.681602
	3					0.950891	0.934605	0.913038
		0.5				0.181775	0.180659	0.178009
		1.0				0.254934	0.253135	0.249113
		1.5				0.453043	0.449290	0.441388
			2			0.295436	0.292247	0.286181
			3			0.307269	0.304604	0.298972
			4			0.315183	0.312792	0.307580
				0.5		0.242323	0.243454	0.218416
				0.9		0.430632	0.442279	0.427574
				1.4		0.451650	0.461434	0.444477
					0.1	0.315832	0.313462	0.308289
					0.3	0.297351	0.295086	0.286581
					0.5	0.242323	0.243454	0.218416

## Conclusions

A theoretical study of the coupled magnetohydrodynamic non-Newtonian Hiemenz plane stagnation flow and heat transfer in a ternary hybrid nanofluid coating under a transverse static magnetic field has been presented. The base fluid (polymeric) considered is engine-oil (EO) doped with graphene $\left(GO\right)$, gold $\left(Au\right)$ and Cobalt oxide $\left(C{o}_{3}{O}_{4}\right)$ nanoparticles. Non-linear radiation, heat source, convective wall heating and magnetic induction effects have been included in the formulation. The Williamson viscoelastic model has been employed to mimic non-Newtonian characteristics, the Rosseland diffusion flux model for radiative transfer and a non-Fourier Cattaneo–Christov heat flux model for thermal relaxation effects. A self-similar non-linear ordinary differential equation boundary value problem has been derived utilizing suitable scaling transformations for the magnetic nanofluid stagnation coating problem. A numerical solution has been developed via RK-4 quadrature in the bvp4c built-in function of MATLAB software. The effects of key control parameters on velocity, induced magnetic field stream function gradient*,* temperature, skin friction, local Nusselt number and streamline (iso-velocity) contours have been visualized graphically and in tables and scrutinized. The relative performance of ternary, hybrid binary and unitary nanofluids for all transport characteristics has also been eval

- Velocity is lowest for the ternary $\mathrm{GO}$–$\mathrm{Au}$–${\mathrm{Co}}_{3}{\mathrm{O}}_{4}$ nanofluid whereas it is highest for the unitary cobalt oxide $\left({\mathrm{Co}}_{3}{\mathrm{O}}_{4}\right)$ nanofluid with increasing magnetic parameter ($\beta ).$
- Temperature and thermal boundary layer thickness is boosted with increment in thermal radiation parameter (*Rd*), heat source parameter (*Q*) and thermal Biot number (*Bi*).
- Streamline magnitudes are reduced in the upper right zone with greater Weissenberg number $$(We)$$ and magnetic Prandtl number ($${Pr}_{m})$$.
- Dimensionless skin friction is significantly greater for the ternary hybrid $GO$–$Au$–$C{o}_{3}{O}_{4}/EO$ nanofluid compared with binary hybrid or unitary nanofluid cases.
- Skin friction (dimensionless surface shear stress at the wall) is reduced with higher values of magnetic parameter $\beta$, Weissenberg number $We$, stretching parameter $A$ and magnetic Prandtl number ${Pr}_{m}$.
- An increment in non-Fourier thermal relaxation time strongly increases temperature and thermal boundary layer thickness.
- Induced magnetic field stream function gradient, $g{^{\prime}}\left(\zeta \right)$ (and the associated magnetic boundary layer thickness) are elevated with magnetic parameter ($\beta$), magnetic Prandtl number $({Pr}_{m}$) and stretching parameter $A$ whereas it is suppressed with increasing Weissenberg number $(We).$

uated. Validation of the MATLAB solutions with previous studies has also been conducted. The principal deductions from the present simulations can be summarized as follows:

The present investigation has explored some interesting aspects of smart functional magnetic nano-coating flows in stagnation materials processing applications. However, Hall current, Ohmic heating and oblique magnetic field effects have been neglected. These are also of interest and may be explored in future studies.

## Data Availability

No data associated in the manuscript.

## List of symbols

${H}_{1}, {H}_{2}$: Magnetic stream function components along $x$-axis and $y$-axis (${\mathrm{ms}}^{-1})$
$u,v$: Velocity components of along $x$-axis and $y$-axis (${\mathrm{ms}}^{-1})$
${v}_{f}$: Kinematic viscosity of the engine oil base fluid (${\mathrm{m}}^{2}{\mathrm{s}}^{-1})$
$${\mu }_{e}$$: Magnetic diffusivity ($${\mathrm{m}}^{2}{\mathrm{s}}^{-1})$$
$x$: Dimensional distance parallel to $x$-axis ($\mathrm{m})$
$y$: Dimensional distance parallel to $y$-axis ($\mathrm{m})$
${\rho }_{thnf}$: Density of ternary nanofluid (${\mathrm{kg m}}^{-3})$
${\mu }_{thnf}$: Dynamic viscosity of ternary nanofluid (${\mathrm{m}}^{2}{\mathrm{s}}^{-1})$
$$T$$: Nanofluid temperature (K)
$\Gamma$: Viscoelastic relaxation time (s)
${k}_{thnf}$: Thermal conductivity of ternary hybrid nanofluid (${\mathrm{Wm}}^{-1}{\mathrm{K}}^{-1})$
${q}_{r}$: Radiative heat flux (W/m^2^)
${Q}_{0}$: Heat source coefficient
${h}_{f}$: Heat transfer coefficient (${\mathrm{Wm}}^{-2}{\mathrm{K}}^{-1})$
$${\alpha }_{thnf}$$: Thermal diffusivity (m^2^/s)
${\varepsilon }_{t}$: Fourier thermal relaxation time (s)
${\left(\rho {c}_{p}\right)}_{thnf}$: Specific heat capacitance of ternary nanofluid ($\mathrm{J kg }{\mathrm{K}}^{-1})$
${\phi }_{1},{\phi }_{2},$ and ${\phi }_{3}$: Volume fractions of graphene oxide $\left(GO\right),$ gold $\left(Au\right)$ and Cobalt oxide $\left(C{o}_{3}{O}_{4}\right)$ nanoparticles (–)
$a$ and$c$: Positive constants (–)
${T}_{\infty }$: Free stream temperature (*K*)
*σ*^∗^: Stefan-Boltzmann radiation constant (5.6704 × 10^−8^ W/m^2^ K)
k*: Extinction coefficient (/Moles cm)
$${N}_{1}-{N}_{5}$$: Constants (–)
$$\zeta$$: Dimensionless variable (–)
$$Bi$$: Biot number (–)
$$We$$: Weissenberg number (–)
$\beta$: Inclined magnetic parameter (–)
$f{^{\prime}}$: Dimensionless velocity function (–)
$g{^{\prime}}$: Dimensionless magnetic flux density (–)
$$\theta$$: Dimensionless temperature (–)
$$Pr$$: Prandtl number (–)
${Pr}_{m}$: Prandtl magnetic number (–)
$Q$: Heat Source parameter (–)
$Rd$: Thermal radiation parameter (–)
${\theta }_{w}$: Temperature ratio parameter (–)
${\Gamma }_{t}$: Thermal relaxation time parameter (–)
$A$: Wall stretching parameter (–)
${Re}_{x}$: Local Reynolds number (–)
${C}_{f}$: Dimensionless friction factor (–)
$$Nu$$: Nusselt number (–)
